# Limitations of PLX3397 as a microglial investigational tool: peripheral and off-target effects dictate the response to inflammation

**DOI:** 10.3389/fimmu.2023.1283711

**Published:** 2023-11-22

**Authors:** Wouter Claeys, Daan Verhaege, Griet Van Imschoot, Elien Van Wonterghem, Lore Van Acker, Laura Amelinck, Federico F. De Ponti, Charlotte Scott, Anja Geerts, Christophe Van Steenkiste, Lien Van Hoecke, Roosmarijn E. Vandenbroucke

**Affiliations:** ^1^ Department of Internal Medicine and Paediatrics, Hepatology Research Unit, Ghent University, Ghent, Belgium; ^2^ Liver Research Center Ghent, Ghent University, Ghent University Hospital, Ghent, Belgium; ^3^ Barriers in Inflammation, VIB-UGent Center for Inflammation Research, VIB, Ghent, Belgium; ^4^ Department of Biomedical Molecular Biology, Ghent University, Ghent, Belgium; ^5^ Laboratory of Myeloid Cell Biology in Tissue Damage and Inflammation, VIB–UGent Center for Inflammation Research, Ghent, Belgium; ^6^ Department of Gastroenterology and Hepatology, Ghent University Hospital, Ghent, Belgium; ^7^ Antwerp University, Department of Gastroenterology and Hepatology, Antwerp, Belgium; ^8^ Department of Gastroenterology and Hepatology, Maria Middelares Hospital, Ghent, Belgium

**Keywords:** microglia, bile duct ligation (BDL), endotoxemia, lipopolysaccharide (LPS), PLX3397, peripheral inflammation, neuroinflammation

## Abstract

Microglia, the resident macrophages of the central nervous system (CNS), play a critical role in CNS homeostasis and neuroinflammation. Pexidartinib (PLX3397), a colony-stimulating factor 1 (CSF1) receptor inhibitor, is widely used to deplete microglia, offering flexible options for both long-term depletion and highly versatile depletion-repopulation cycles. However, the potential impact of PLX3397 on peripheral (immune) cells remains controversial. Until now, the microglia-specificity of this type of compounds has not been thoroughly evaluated, particularly in the context of peripherally derived neuroinflammation. Our study addresses this gap by examining the effects of PLX3397 on immune cells in the brain, liver, circulation and bone marrow, both in homeostasis and systemic inflammation models. Intriguingly, we demonstrate that PLX3397 treatment not only influences the levels of tissue-resident macrophages, but also affects circulating and bone marrow immune cells beyond the mononuclear phagocyte system (MPS). These alterations in peripheral immune cells disrupt the response to systemic inflammation, consequently impacting the phenotype irrespective of microglial depletion. Furthermore, we observed that a lower dose of PLX3397, which does not deplete microglia, demonstrates similar (non-)MPS effects, both in the periphery and the brain, but fails to fully replicate the peripheral alterations seen in the higher doses, questioning lower doses as a ‘peripheral control’ strategy. Overall, our data highlight the need for caution when interpreting studies employing this compound, as it may not be suitable for specific investigation of microglial function in the presence of systemic inflammation.

## Graphical Abstract

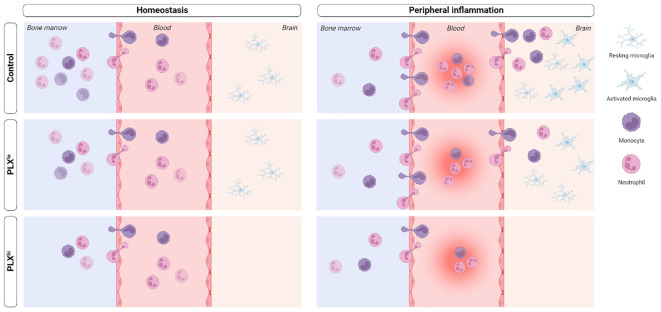

## Introduction

Tissue resident macrophages, monocytes, classical dendritic cells (cDCs) and their progenitors together form the mononuclear phagocyte system (MPS). Macrophages are specialized tissue phagocytes derived from embryonic progenitors, including yolk-sac macrophages and fetal liver monocytes (1–3). Macrophage proliferation and differentiation is controlled by signals from the colony-stimulating factor 1 receptor (CSF1R) (4). In the brain, parenchymal microglia are the main macrophage population. Microglia develop from yolk-sac precursors in a CSF1R dependent manner (5). In steady state conditions, they continuously scan their environment in search of pathogens or tissue damage (6–8). In disease conditions, microglia develop a so-called activated phenotype, accompanied by an amoeboid morphology and increased cyto/chemokine production (7). Often, this is associated with a significant influx of bone marrow-derived monocytes (8–11).

To investigate the role of microglia in homeostasis and disease, various microglial depletion strategies have been developed (reviewed in (12)). As microglia are thought to be particularly dependent on CSF1R signaling, many of these strategies target this receptor (13, 14). However, due to the general importance of CSF1R in macrophage biology, full body knockout strategies were plagued by a shortened lifespan, developmental deficits (15) and reduced tissue macrophage levels beyond the brain (15, 16). Inducible genetic depletion models on the other hand only allowed for short term microglial depletion (12). The development of blood-brain barrier (BBB) penetrant, small molecule CSF1R kinase inhibitors (CSF1Ri), initially PLX3397 (17) and later PLX5622 (18), has revolutionized the field of microglia research. Using these compounds, long-term microglial depletion can be achieved without evident side effects. Furthermore, due to the reversible nature of the inhibition, highly flexible microglial depletion and repopulation cycles can be achieved. A considerable body of literature emerged, showing that the effect of BBB penetrant CSF1Ri treatment is highly dependent on the specific disease context and the type of modulation employed, such as repopulation paradigms *versus* continued depletion (8, 19, 20).

In line with studies involving full-body knockout mice and CSF1R blocking antibodies (21), it was claimed that PLX3397/PLX5622 had no significant effects on hematopoiesis and circulating immune cells (18, 22–24). Additionally, it was hypothesized that, compared to other tissue macrophages, microglia may be particularly dependent on CSF1R for their survival, based on the extremely low brain concentrations of CSF1Ri needed to achieve complete microglial depletion (17, 18, 21). To control for potential off-target effects on peripheral macrophages, some authors suggested the inclusion of a control group treated with a lower dose CSF1Ri that has minimal central nervous system (CNS) presence and neglectable impact on microglia, although this strategy has scarcely been implemented (12, 17, 18). However, whether lower doses have similar peripheral effects despite vastly different plasma concentrations compared to the higher dose has not been studied. Additionally, the proposed control dose of PLX3397 [75 ppm in chow] can still be detected in brain tissue, raising the question whether CNS effects at this dose are really negligible (17, 18). Finally, subsequent studies challenged the notion that PLX3397 and PLX5622 have a negligible impact on peripheral tissue macrophages and especially circulating cells and bone marrow cells, some of which even do not express CSF1R, e.g. CD4^+^ T-cells (25–31).

Despite the prior belief that the brain is an ‘immune-privileged’ site, peripheral inflammation is now known to play a pivotal role in neuropathology, including *via* direct immune cell infiltration into the brain (8, 32, 33). In light of this periphery-brain communication, the potential non-microglial effects associated with BBB-penetrant CSF1Ri PLX3397 and PLX5622 have either been insufficiently explored or disregarded, possibly explaining the the sometimes very divergent results when employing different kinds of CSF1R modulation in similar disease contexts. To illustrate, in the 5xFAD model of Alzheimer’s disease, pharmacological microglial depletion and constitutive absence in Csf1r^ΔFIRE/ΔFIRE^ mice produce a quite divergent phenotype (18, 34). Recent studies using PLX5622 have demonstrated that this compound prolongs sickness behaviour after lipopolysaccharide (LPS) injection, ameliorates Western Nile virus encephalitis, and induces massive mortality in polymicrobial sepsis (31, 35, 36). The specific role of microglial depletion, compared to effects on peripheral MPS cells, was either limited or absent (35, 36). Furthermore, evidence suggests that the improved phenotype in Western Nile virus encephalitis may be directly attributed to effects of PLX5622 on bone marrow production of monocytes. While off-target effects of PLX3397 have been reported under steady state conditions (29), its impact in the context of peripherally-derived neuroinflammation remains unexplored.

Motivated by these observations, our study aimed to investigate the influence of PLX3397 treatment on the response to a peripheral inflammatory stimulus, with or without microglial presence. Our findings demonstrate that PLX3397 treatment significantly affects the response to acute and chronic inflammatory stimuli, associated with direct effects on the mature myeloid cell pool in the circulation and bone marrow, including cells not expressing CSF1R. Surprisingly, non-depleting doses of PLX3397 exhibit similar off-target toxicity, but still affect the brain, questioning its use as a ‘peripheral control’. These findings shed light on the complexities surrounding PLX3397 and its effects on the peripheral inflammatory response and circulating immune cells, highlighting the need for thorough consideration of its off-target effects when employing this compound.

## Materials and methods

### Animals

10 to 15-week-old male C57Bl/6j wild type (Janvier Labs, Le Genest-Saint-Isle, France) were used. Mice were housed with 14-to-10-hour light and dark cycles and free access to food and water in specific pathogen free (SPF) conditions. All experiments complied with the current laws of Belgium (Law of 14. August 1986 related to protection and welfare of animals) and EU directive 2010/63/EU, and were approved by the animal ethics committee of the Faculty of Sciences, Ghent University (EC 2022-070, EC2023-034, EC2023-018).

### Microglial depletion

PLX3997 was provided by Medchemexpress (HY-16749). It was formulated in standard AIN-76A rodent chow at a concentration of 75 ppm (PLX^lo^) and 600 ppm (PLX^hi^) (Research Diets, New Brunswick, NJ) and provided *ad libitum*. PLX3397 diet was administered for 7 (steady state/LPS experiments) or 9 (BDL experiments) days consecutively. Control mice were fed withs standard AIN-76A diet *ad libitum*. Average daily food intake and average ingested PLX dose was assessed after 7 days of diet. Body weight was assessed daily.

### Motor function assessment

Motor function tests were performed 2 weeks before (baseline) and 6 days after diet start. Two tests (difficult beam traversal test and open field test (OFT)) were carried out to gain insights into motor function and exploratory behaviour. In short, in the OFT, mice were allowed to move freely in a clear open-field area (40x40x40 cm) for 5 min. Travelling distance, a measure of general activity, as well as time spent in the center and border zones of the open field, a measure of anxiety, were assessed. For the difficult beam traversal, mice were trained to traverse a beam of 1m with 4 sections of narrowing width (3.5, 2.5, 1.5 and 0.5 cm) placed on top of 2 inverted mouse cages. During the testing phase, a metal grid was placed on top of the beam. Time to traverse the beam was measured. Detailed protocols can be found in **supplementary file 1**. All tests were video recorded. To exclude the existence of olfactory clues, all objects were thoroughly cleaned with 20% ethanol after each trial. Analysis of OFT was performed using Ethovision XT 15 software (Noldus).

### Models of peripheral inflammation

LPS from *S. abortus equi* (L-5886) was obtained from Sigma-Aldrich and diluted in sterile phosphate bufferred saline (PBS). For survival experiments, LPS was injected intraperitoneally (IP) at an LD_50_ dose (5.0 mg/kg). Body weight, temperature and mortality were recorded every 2 hours for the first 12 hours and 3 times per day afterwards. For all other experiments, LPS was injected IP at an LD_0_ dose (2.5 mg/kg). Body weight and temperature were followed up every 6 h. Mice were sacrificed 6 and 24 hours after injection. Control mice received an equivalent volume of sterile PBS and were sacrificed 24 hours after injection.

The bile duct ligation (BDL) and sham procedures were performed under sterile conditions as previously described (37). In short, under isoflurane inhalation anesthesia, a midline abdominal incision was made and the common bile duct was isolated from the flanking portal vein. Next, the common bile duct was occluded with a double ligature of a non-resorbable suture (Mersilk 5-0, Ethicon 682H) and cut in between ligatures to prevent recanalization. Mice received buprenorphine 0.1 mg/kg intraperitoneally for 72 hours to prevent postoperative pain and distress (38) and mice were sacrificed 14 days post-surgery. The study is reported in accordance with ARRIVE guidelines (39).

### Tissue sample collection

Mice were sedated through IP injection with an overdose of ketamine (87.5 mg/kg) and xylazine (12.5 mg/kg). After disappearance of paw and tail reflexes, whole blood was isolated using heart puncture, collected in Microvette 500 K2 ethylenediaminetetraacetic acid (EDTA) tubes (Sarstedt, 20.1339.100) and stored at 4°C until analysis. Afterwards, mice were transcardially perfused using 10 ml 0.2% heparin (Sigma, H‐3125) in ice-cold D‐PBS (Gibco, 14190‐094) per mouse (4.50 ml/min), followed by brain, cerebrospinal fluid (CSF) and liver sample isolation.

### Analysis of BBB and blood-CSF barrier integrity

Fifteen minutes before sacrifice, mice were injected intravenously (IV) with 250 mg/kg FITC-labelled dextran (4 kDa, Sigma). Mice were perfused with ice-cold D-PBS/heparin (0.2% heparin (5000 IU/ml, Wockhardt) to remove blood. Adequate perfusion was checked visually. CSF was obtained from the fourth ventricle *via* the cisterna magna puncture method as described previously (40). Briefly, borosilicate glass capillary tubes (Sutter Instruments, B100-75-15) were used to pull needles on the Sutter P-87 flaming micropipette puller (pressure 330 Pa, heat index 300). The incision site was sterilized with 70% ethanol. The cisterna magna was exposed by dissecting skin and muscle tissue on the posterior side of the skull. The head of the mouse was mounted at an angle of 135°, and CSF was collected from the fourth ventricle by puncturing the cisterna magna using the capillary needles. Samples were centrifuged for 5 min at 300 g at 4°C to assess blood contamination. To measure blood-CSF barrier leakage, CSF was diluted 1/100 in PBS and fluorescence was measured. Brain samples were lysed mechanically and subsequently incubated overnight at 37°C in formamide (47671; Sigma-Aldrich) while shaking and in the dark. The next day, 100 µl supernatant was collected after centrifugation at 400 g for 7 min and fluorescence level was assessed, (λ_ex_/λ_em_ = 485/520 nm) using the FLUOstar omega (Isogen LifeScience). Weight-corrected tissue fluorescence was used to determine BBB integrity.

### Complete blood cell count analysis

EDTA-anticoagulated blood was kept on ice and complete blood cell count analysis was performed using the Vetscan HM5 Hematology Analyzer (Zoetis) according to the manufacturer’s instructions within 12 hours after isolation.

### Plasma biomarker measurement

Plasma was isolated from whole blood by centrifugation and isolation of supernatant (10 min, 1300 g, 4°C followed by 15 min, 2400 g, 3°C). Plasma levels of alanine aminotransferase (ALT) and bilirubin were assessed using the Architect c16000 (Abbott). Plasma endotoxin levels were assessed using the mouse LPS ELISA kit (Cusabio, CSB-E13066m) according to the manufacturer’s instructions or the Limulus Amebocyte Lysate (LAL) assay (Associates of Cape Cod Inc.) according to the manufacturer’s instructions, depending on experiments.

### Isolation of brain cells

For astrocyte and microglial isolation, whole brain samples were collected in ice-cold in 1 x HBSS -/- and cut to pieces approximately 1 mm³ in size using spring scissors. Brain slurry was dissociated into single cell suspensions using the Neural Tissue Dissociation Kit (P) (Miltenyi Biotec, 130‐092‐628) as described previously (41). In short, cells were enzymatically dissociated using activated enzyme (P) for 15 min at 37°C and enzyme (A) for 2x 10 min at 37°C under continuous nutation. Additionally, the samples were mechanically dissociated by trituration 10 times with a 5 ml serological pipet in between enzymatic dissociation steps. To stop the enzymatic reaction, samples were diluted with an excess of HBSS -/-. The samples were then passed through a 70 µM cell strainer (BD Falcon, 734‐0003) and mixed with 90% Percoll™ PLUS (equilibrated in HBSS‐/‐, pH7.4; Merck, GE17‐5445‐02) and DNase I amplification grade (10U/µl; Invitrogen, 18068‐015) to obtain a final concentration of 24% Percoll™ PLUS and 75U/ml DNase. Following, the samples were spun down at 300 g for 11 min at room temperature (RT) with a low acceleration and deceleration brake. The myelin layer and supernatans were aspirated and the pellet was resuspended in 50 µl of 0.5% BSA (Jackson ImmunoResearch, 001‐000‐162) in D‐PBS (Gibco 14190‐094). This process was repeated twice. Single cell suspensions were kept on ice until staining and cell sorting.

For immune cell phenotyping, the right brain hemisphere was collected in 1 ml of ice-cold RPMI 1640 (Gibco, 52400-025) and cut to pieces approximately 1 mm³ in size using spring scissors. Brain slurry was enzymatically dissociated as previously described (41). In short, brain halves were incubated with DNase I, collagenase I and collagenase IV at a final concentration of 30 U/ml, 10 U/ml and 400 U/ml respectively, for 30 min at 37°C, with mechanical dissociation using a p1000 micropipette every 10 min. Samples were passed twice through a 70 µM cell strainer (BD Falcon, 734‐0003), centrifuged and supernatant was removed. Cell pellets were resuspended in 10 ml of 30% Percoll™ PLUS (equilibrated in HBSS‐/‐, pH7.4; Merck, GE17‐5445‐02) and spun down at 600 g for 10 min at 4°C, with no acceleration and break. Myelin layers and supernatant were removed, and pellets were resuspended in FACS buffer (2 mM EDTA, 2% BSA in 1x HBSS -/-). Suspensions were kept on ice until staining and flow cytometry.

### Isolation of bone marrow cells

For bone marrow isolation, one hind leg was collected in ice-cold RPMI. After thorough removal of skin and muscle tissue, knee and hip joints were cut from tibia and femur respectively, and bone marrow cells were isolated by centrifugation (1900 g, 1 min, RT). Samples were filtered over a 70 µm mesh (BD Falcon, 734‐0003) and red blood cells were lysed using ACK lysis (Westburg, 10-548E) for 1 min at RT. The reaction was stopped with an excess of 1x PBS, cells were centrifuged (400 g, 7 min, 4°C) and kept on ice before proceeding to antibody staining and flow cytometry.

### Isolation of liver cells

Liver cells were isolated by liver perfusion and digestion as described previously (26). Briefly, after retrograde cannulation, livers were perfused for 1-2 min with an EGTA-containing solution, followed by a 5 min (6 ml/min) perfusion with 0.2 mg/ml collagenase A. Livers were then removed, minced and incubated for 20mins with 0.4 mg/ml collagenase A and 10 U/ml DNase at 37°C. All subsequent procedures were performed at 4°C. Samples were filtered over a 100 µm mesh filter and red blood cells were lysed. Samples were again filtered over a 40 µm mesh filter. After two centrifugation steps of 1 min at 50 g to isolate hepatocytes, remaining liver cells (leukocytes, LSECs and HSCs) were centrifuged at 400 g for 5 min before proceeding to antibody staining for flow cytometry.

### Flow cytometry and cell sorting

For isolation of brain and BM cells, single cell suspensions were pre-incubated with Fc Block (1/100; BD Biosciences, 553142) for 10 min at 4°C and stained with appropriate antibodies at 4°C in the dark for 30 min. For isolation of liver cells 0.5-5x10 (6) cells were pre-incubated with 2.4G2 antibody (Bioceros) to block Fc receptors and stained with appropriate antibodies at 4°C in the dark for 30-45 min. Antibodies and dilutions are listed in **Supplementary Table 1**. Reactions were stopped by adding an excess of staining buffer, cells were spun down at 400 g for 7 min at 4°C. Pellets were resuspended in FACS buffer and transferred through a 35 µm mesh into a 5 ml Falco^®^Round‐Bottom Polystyrene Test Tubes with Cell Strainer Snap Cap (Fisher Scientific, 08‐771‐23). Cell viability was assessed using a Fixable Viability dye (eFluor506; Thermo Fisher, 65-0866-14).

Flow cytometry and cell sorting was performed on the BD FACSymphony S6. Cells were sorted into 2 ml eppendorfs containing 450 µl RLT Plus lysis buffer (QIAGEN) containing 1% BME. The samples were extensively vortexed and stored at ‐80°C until RNA isolation. Flow cytometry without cell sorting was performed on the BD FACSymphony A3 or A5. Flow cytometry plots were analyzed using FlowJo 10.8.1. Gating strategies for astrocyte isolation as well as brain, bone marrow and liver immune phenotyping can be found in **Supplementary figure 1**, **2** and **3** respectively.

### RNA isolation and real-time qPCR

Selected brain regions (cortex, hippocampus) and liver tissue were isolated, snap frozen in liquid nitrogen and stored at -80°C until analysis. Liver samples for RNA analyses were submerged in an excess of RNA Later (Thermo Fischer Scientific) and kept overnight at 4°C, after which they were transferred to -80°C until further analysis.

Tissue RNA was isolated using the Aurum total RNA Mini Kit (Bio-Rad), according to the manufacturer’s instructions. RNA concentration and purity were determined spectrophotometrically using the Nanodrop ND-1000 (Nanodrop Technologies, Thermo Scientific). RNA from sorted cells was isolated using the RNeasy ^®^Plus Micro Kit (QIAGEN, 74034) according to the manufacturer’s instructions. The concentration and purity of the RNA was determined using the Agilent RNA 6000 Pico Kit (Agilent 5067-1513) and the Agilent 2100 Bio-Analyzer. A RIN value of 7 or higher was required to proceed. cDNA was synthesized with the SensiFAST™ cDNA Synthesis Kit (Bioline). Real time-qPCR was performed with the Light Cycler 480 system (Roche) using SensiFAST SYBR No-Rox (Bio-Line). Volumes were dispensed using the I.DOT (DISPENDIX). Expression levels were normalized to the expression of all stable (at least two) reference genes, determined using the geNorm Housekeeping Gene Selection Software (42). Sequences of forward and reverse primers can be found in **Supplementary Table 2**.

### Histology

For microscopy analyses, liver and brain tissue fixed in 4% PFA overnight at 4°C. After dehydration steps, samples were embedded in paraffin at RT until further use.

Five µm liver tissue sections were cut (HM 340 E, Thermo Fischer Scientific) and stained with haematoxylin-eosin (Klinipath). Sections were imaged using Zeiss Axioscan Z.1 (x10 magnification, Zeiss, Germany). All images were stained simultaneously and evaluated in a blinded manner. Bile infarcts fractions were quantified using ImageJ (version 1.53c, National Institutes of Health). Images of at least 10 random low power fields were analyzed per liver section. Bile infarcts were quantified on H&E sections as a percentage necrotic are relative to the total section area.

### Confocal microscopy

Brain samples were cut into 5 μm paraffin sections (HM 340 E, Thermo Scientific). Sections were permeabilized in PBS containing 0.5% Triton X-100. Following blocking with blocking buffer (5% species matched serum, 0.5% Triton X-100 in PBS) at RT for 1 h, sections were incubated with primary antibodies in blocking buffer at 4°C overnight. Serum was matched to species of secondary antibodies. The same blocking buffer was used to dilute primary and secondary antibodies. After washing with PBS, sections were stained with fluorophore-conjugated secondary antibodies in blocking buffer at RT for 2 h. The full list of antibodies can be found in **Supplementary Table 3**. Counterstaining was done with DAPI 1/1000 in PBS. Confocal images were taken with a Zeiss LSM 780 (Zeiss, Germany), using a Plan-Apochromat 40 × 1.3 oil DIC UV-IR M27 objective or a Plan-Apochromat 25x 0.8 Imm Korr DIC M27 objective. Image analysis was performed using ImageJ (version 1.53c, National Institutes of Health).

### Statistics

For comparison of two groups, unpaired t-test or Mann-Whitney test were used based on normality testing of data. For comparison of multiple groups, significance was determined using one-way ANOVA with Dunnett *post-hoc* testing (comparison with a single control group), Sidak *post-hoc* testing (comparison between all groups) or Kruskal-Wallis with Dunn *post-hoc* testing, based on normality distribution of residuals. For comparison of grouped data, 2-way ANOVA with Dunnett *post-hoc* testing was performed. ANOVA tables can be found in **Supplementary Tables 4**–**9** PLX^lo^ and PLX^hi^ groups were only compared with AIN76A chow fed animals. For survival analysis, survival curves were compared using the log-rank test with Holm-Sidak multiple testing correction. The logrank test for trend was used to evaluate effect of different doses of PLX3397 on survival. All testing was two-sided. Differences were considered significant at p<0.05. * p<0.05, ** p<0.01, *** p<0.001, **** p<0.0001. All data is represented as mean + SEM. Graphpad 9.2 (LaJolla, California) was used for all statistical analyses.

## Results

### A high dose of PLX depletes microglia and peripheral macrophages without affecting tissue and circulating monocyte levels

**Figure 1:**
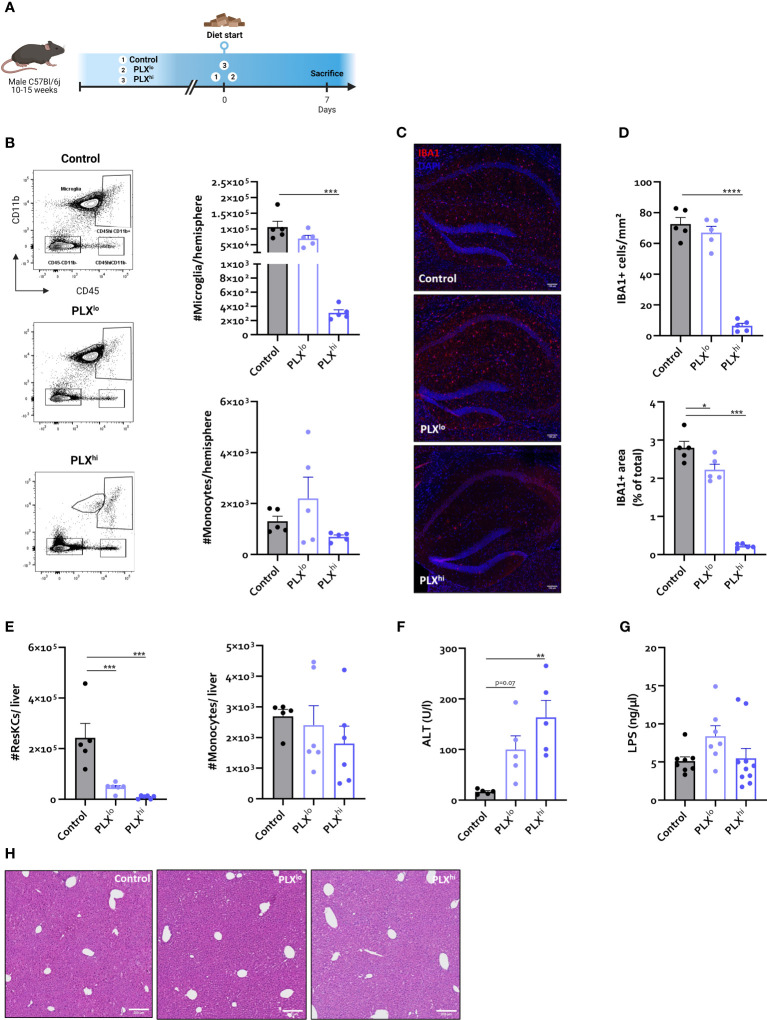
In steady state conditions. PLX3397 has dose dependent effects on tissue macrophages without affecting monocyte levels. **(A)** Experimental set-up. Mice were treated with AIN76A chow containing no (control), low (75 ppm; PLX^lo^) or high (600 ppm; PLX^hi^) concentrations of PLX3397 for 7 days prior to sacrifice and analysis. **(B)** Representative flow cytometry plots and absolute cell count of microglia and Ly6C^+^ monocytes per hemisphere. **(C–D)** Representative images of IBA1 staining in hippocampus **(C)** and quantification of IBA1+ cell number and stained area **(D)**. Scale bar represents 100 µm. Data are derived from a single experiment with n=5/group. **(E, F)** Absolute cell count of resident Kupffer cells and Ly6C^+^ monocytes in mouse liver **(E)** and plasma ALT levels **(F)**. Data are derived from a single experiment with n=5-6/group. **(G)** Plasma LPS levels. Data is derived from 2 independent repeats with n=7-10/group. **(H)** Representative H&E images of liver tissue. Scale bar represents 200 µm. * p<0.05, ** p<0.01, *** p<0.001, **** p<0.0001.

We utilized PLX3397 mixed in AIN76A chow at two different doses: PLX^hi^ (600 ppm), a dose reported to deplete microglia, and PLX^lo^ (75 ppm), a dose that preserves microglial cell numbers (17). Chow containing the PLX dose was provided *ad libitum* for 7 days (**Figure 1A**). On average, daily PLX3397 intake was ~59 mg/kg body weight in the PLX^hi^ group and ~8.5 mg/kg body weight in the PLX^lo^ group. We observed weight loss in the PLX^hi^ group, totalling 5% after 7 days (**Figure S4A**). This weight loss was likely due to reduced food intake (**Figure S4B**), and is in line with what is reported in conditional microglial depletion in rats (43). Additionally, in PLX^hi^ mice, a slight reduction in activity in the open field was observed, which was not associated with anxiety-like behaviours nor apparent impairment in balance function (**Figures S4C, D**).

As expected, flow cytometry confirmed ablation of >99% of all microglia within 7 days after PLX^hi^ treatment, while PLX^lo^ chow did not significantly affect microglial numbers. Brain monocyte numbers were not affected by either dose (**Figure 1B**). These results were confirmed on hippocampal ionized calcium-binding adapter molecule 1 (IBA1) immunofluorescence staining. Despite similar numbers of microglia, we observed that IBA1+ stained area was slightly reduced in PLX^lo^ treated animals compared to controls (**Figure 1D**).

**Figure 2:**
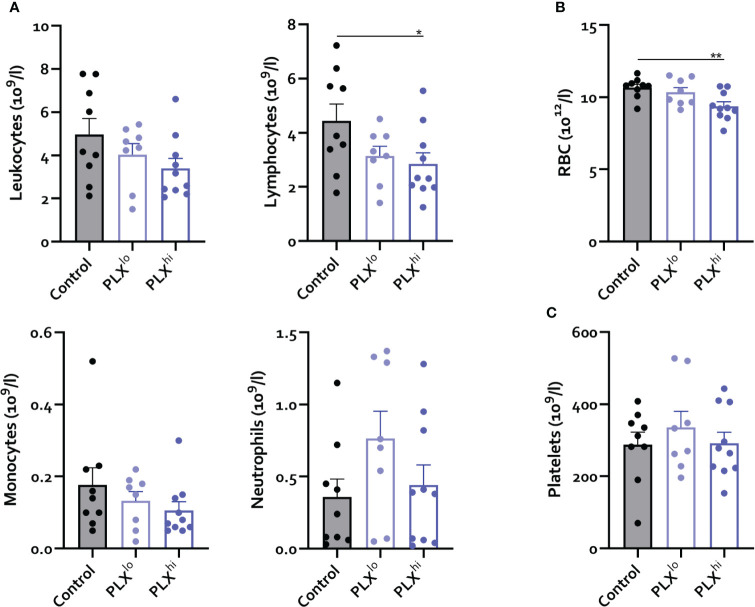
PLX3397 does not affect the levels of circulating monocytes. Mice were treated with AIN76A chow containing no (control), low (75 ppm; PLX^lo^) or high (600 ppm; PLX^hi^) concentrations of PLX3397 for 7 days prior to sacrifice and analysis. **(A–C)** Levels of circulating leukocytes, monocytes, neutrophils, lymphocytes **(A)**, red blood cells **(B)** and platelets **(C)**. Data is derived from 2 independent experiments with n=8-10/group. * p<0.05, ** p<0.01, *** p<0.001, **** p<0.0001.

Given the dependence of non-CNS tissue macrophages on CSF1R for survival and the reported association of PLX3397 with liver injury in humans (44), we assessed levels of resident Kupffer cells (KCs), the liver resident macrophages, after PLX treatment. Both doses significantly reduced KC levels. Again, this was not associated with changes in tissue monocyte levels (**Figure 1E**). Although increased plasma ALT levels were detected, indicating potential liver damage, KC depletion did not lead to apparent liver injury on H&E staining. Furthermore, KC reductions did not affect hepatic bacterial clearance, based on similar plasma LPS levels in all treatment groups (**Figure 1G, I**). It has been reported that depleted microglia are not replaced by infiltrating monocytes (45), but the opposite is true for KCs (26, 46). We performed complete blood count analysis (CBC) to assess whether decreased circulating monocyte levels could account for this lack of infiltrating liver monocytes despite KC depletion. This did not reveal significant differences in levels of circulating monocytes, neutrophils or platelets. However, PLX^hi^ but not PLX^lo^ chow feeding induced a slight but significant decrease in blood lymphocyte and red blood cell (RBC) levels (**Figure 2A, B**).

As we observed effects of PLX3397 treatment beyond microglia outside of the brain, we sought to investigate whether central nervous system (CNS) effects of PLX3397 were specific to microglia. Consistent with previous findings (17), a microglia-depleting dose of PLX3397 induced upregulation of the astrocyte reactivation marker glial fibrillary acidic protein (*Gfap*) in sorted astrocytes without affecting astrocyte number or GFAP immunoreactivity on staining (**Figure S5A-E**). Strikingly, this effect was also evident in PLX^lo^ treated animals. On the other hand, oligodendrocyte and neuron staining did not reveal differences in cell number and stained area between groups (**Figure S5F-I**). We confirmed that PLX3397, at least when given in low doses, did not induce changes in blood-brain barrier (BBB) permeability. Nevertheless, we found that PLX^lo^ treated mice exhibited a disturbed blood-cerebrospinal fluid (CSF) barrier, which is made up by epithelial cells in the choroid plexus (ChP), a region devoid of microglia (**Figure S5J**) (47).

### PLX treatment increases mortality in severe peripheral inflammation

**Figure 3:**
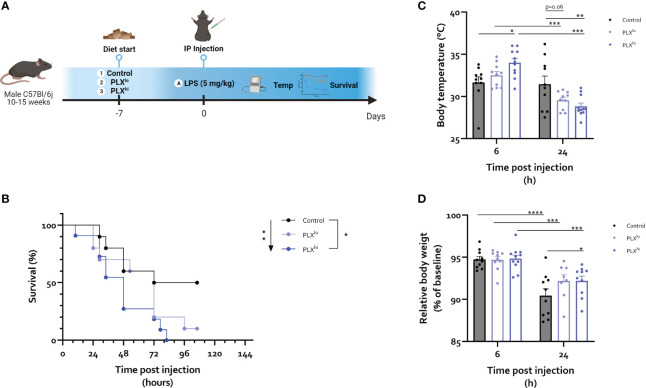
PLX3397 exacerbates the response to an LD_50_ dose of LPS. **(A)** Experimental set-up. Mice were treated with AIN76A chow containing no (control), low (75 ppm; PLX^lo^) or high (600 ppm; PLX^hi^) concentrations of PLX3397 for 7 days prior to injection of LPS (5 mg/kg, IP). Survival, body weight and temperature were followed up. **(B)** Survival after LPS injections. Arrow indicates logrank test for trend, bar indicates log-rank test comparing individual groups. **(C, D)** Body temperature **(C)** and relative body weight **(D)** 6 and 24 hours after LPS injection. Data from a single repeat with n=10-11/group. * p<0.05, ** p<0.01, *** p<0.001, **** p<0.0001.

Based on the observed effects of PLX3397 on tissue resident macrophages in brain and liver and the absence of effects on circulating immune cells, we next investigated if PLX3397-treated mice respond differently to an LD_50_ dose of lipopolysaccharide (LPS) (**Figure 3A**). This endotoxemia model mimics the severe inflammatory response syndrome in sepsis without the presence of microbial infection (48). Interestingly, mortality rates were significantly increased in mice pretreated with PLX^hi^. The PLX^lo^ pretreated mice showed an intermediary phenotype, albeit not significantly different from control chow treated animals. Nonetheless, when performing the log-rank test for trend, increasing mortality was found with increasing doses of PLX3397 (**Figure 3B**). Additionally, the LPS-induced temperature drop was both delayed and exacerbated in PLX^hi^ treated mice compared to control chow fed mice, with higher body temperatures at 6 hours, but lower at 24 hours post LPS injection. Again, PLX^lo^ treated mice exhibited an intermediary phenotype, with a trend towards lower body temperatures compared to control chow fed mice 24 hours after LPS. Only in PLX3397 pretreated mice, we observed significant differences in body temperature between the two timepoints (**Figure 3C**). The body weight drop 24 hours after LPS was slightly lower in PLX treated groups (**Figure 3D**).

**Figure 4:**
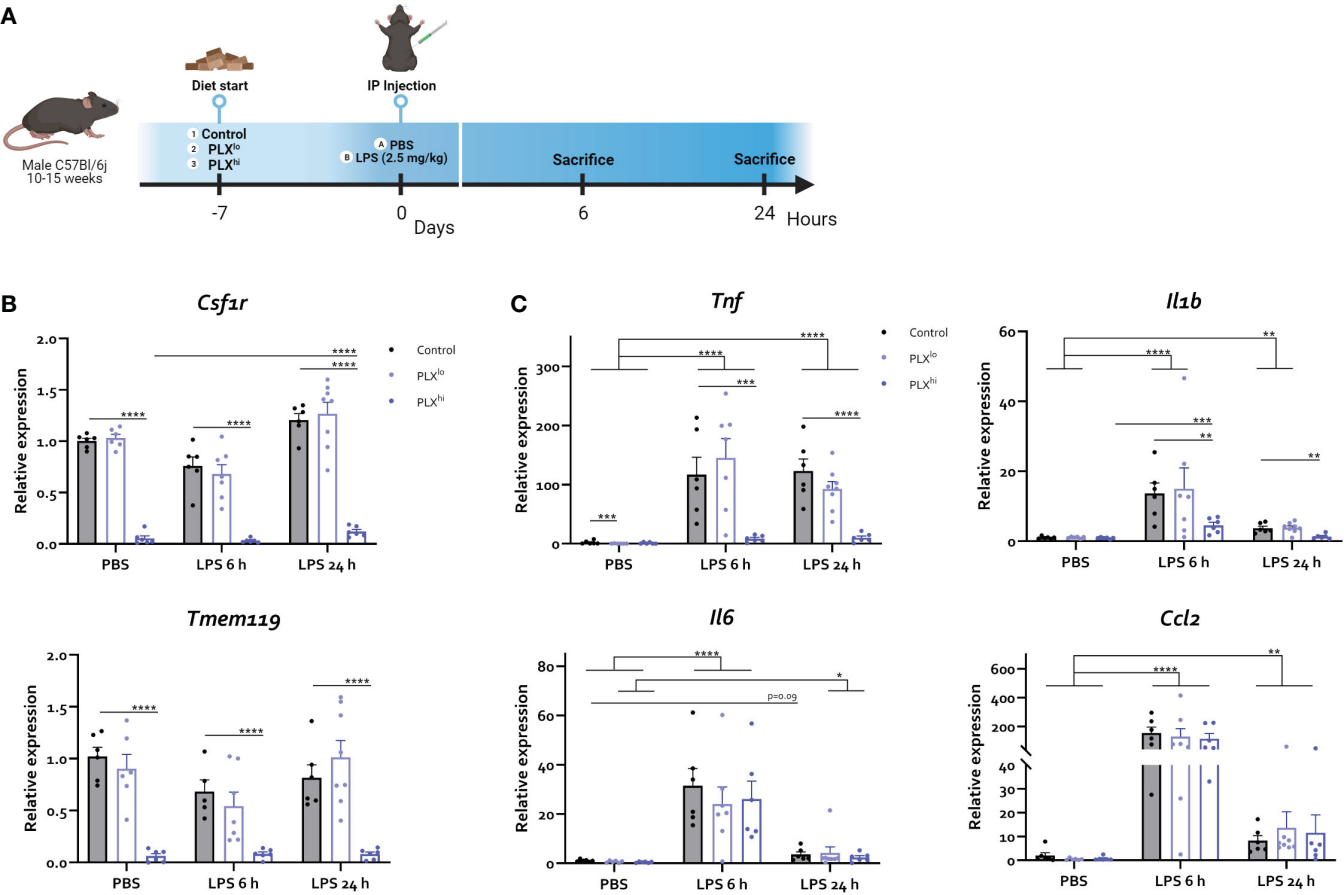
Microglial depletion partially abrogates the cerebral inflammatory response. **(A)** Experimental set-up. Mice were treated with AIN76A chow containing no (control), low (75 ppm; PLX^lo^) or high (600 ppm; PLX^hi^) concentrations of PLX3397 for 7 days prior to injection of LPS (2.5 mg/kg, IP). Mice were sacrificed 6 and 24 hours after injection. **(B, C)** Hippocampal mRNA levels of the microglia/macrophage markers *Csf1r*, *Tmem119* **(B)** and the inflammatory cytokines *Tnf, Il1b*, *Il6* and chemokines *Ccl2* **(C)**. All data are derived from 4 independent repeats with n=8/group. * p<0.05, ** p<0.01, *** p<0.001, **** p<0.0001.

Next, we shifted to an LD_0_ of LPS (2.5 mg/kg IP) to investigate the mechanisms of this PLX3397-induced increased sensitivity for LPS in more detail (**Figure 4A**). This did not lead to mortality within 24 hours in any treatment group. Although no significant differences were observed between treatment groups, temperature differences following LPS followed a similar pattern to the LD_50_ dose experiments (**Figure S6**). To examine whether alterations in the cerebral inflammatory response could account for the aggravated phenotype, brain cyto/chemokine responses were evaluated. This revealed a significant decrease in microglia/macrophage markers *Tmem119* and *Csf1r* in the PLX^hi^ pretreated mice, both after LPS and PBS injection (**Figure 4B** **;** **Table S4**). Additionally, LPS-associated increases in *Tnf* and *Il1b*, but not *Il6* and *Ccl2*, gene expression level were abrogated by PLX^hi^ pretreatment (**Figure 4C** **;** **Table S4**). PLX^lo^ treatment had no effect on transcription of microglia/macrophage marker genes nor cytokine or chemokine mRNA levels, suggesting an altered cerebral cytokine response does not explain the aggravated response to LPS at this dose.

We wanted to assess whether PLX3397 chow treatment after the primary inflammatory stimulus in a more chronic inflammation model would induce similar results. Considering the effect we observed on liver resident macrophages and the development of transaminase elevations in patients treated with pexidartinib (44), we opted for the BDL model, an archetypical model of inflammatory liver injury associated with secondary microglial activation and hepatic encephalopathy (38, 41, 49). PLX3397 chow treatment was commenced 5 days after BDL, until sacrifice at 14 days (**Figure S7A**). Mortality was only observed in PLX-treated mice (PLX^lo^: 20% at 14 days; PLX^hi^: 29% at 14 days) (**Figure S7B**) and BDL mice treated with PLX^hi^ showed a more pronounced temperature drop (**Figure S7C**).

### Exacerbation of liver injury after PLX3397 treatment is dependent on dose and model system

**Figure 5:**
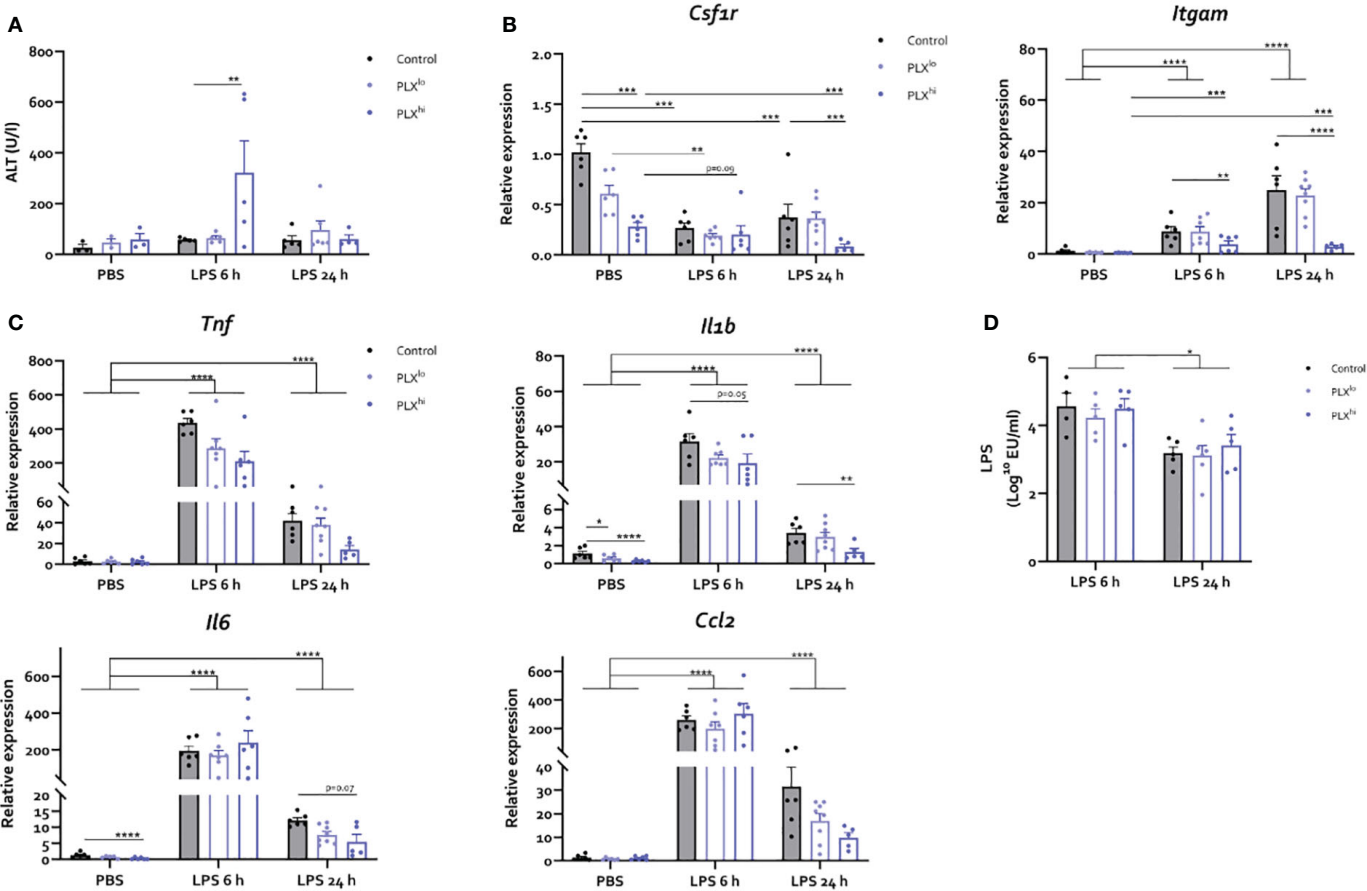
PLX3397 has limited and dose specific effects on the hepatic response to LPS. Mice were treated with AIN76A chow containing no (control), low (75 ppm; PLX^lo^) or high (600 ppm; PLX^hi^) concentrations of PLX3397 for 7 days prior to injection of LPS (2.5 mg/kg, IP). Mice were sacrificed 6 and 24 hours after injection. **(A)** Plasma ALT levels. **(B, C)** Liver mRNA levels of the macrophage marker *Csf1r*, the myeloid marker *Itgam* **(B)**, the inflammatory cytokines *Tnf, Il1b*, and *Il6* and the chemokine *Ccl2* **(C)**. **(D)** Plasma endotoxin levels after LPS injection. All data are derived from 4 independent repeats with n=3-8/group. * p<0.05, ** p<0.01, *** p<0.001, **** p<0.0001.

Next, we studied whether the heightened sensitivity of PLX3397-treated mice to systemic inflammation could be attributed to its effects on KCs and/or subsequent altered hepatic response, which (partly) dictates the systemic and neuroinflammatory response in both the BDL and LPS models. However, in the LPS endotoxemia model, we observed only limited effects of PLX3397 pretreatment on the hepatic response and these effects were unique to the PLX^hi^ pretreated group. Specifically, we found a transient increase in ALT levels 6 hours after LPS, only in the PLX^hi^ group (**Figure 5A** **;** **Table S7**). Bilirubin levels were below the detection limit suggesting liver function was preserved in all mice (data not shown). Hepatic mRNA levels of the macrophage marker *Csf1r* levels decreased after LPS, while myeloid marker *Itgam* transcription increased. These changes were ablated in PLX^hi^ treated mice, while levels in PLX^lo^ treated animals were comparable with control conditions. Of note, an intermediary reduction of *Csf1r* mRNA levels in PLX^lo^ treated animals was observed in PBS injected animals (**Figure 5D** **;** **Table S5**). Regarding the cytokine response, we observed (non significant) reductions in levels of *Tnf* and *Il1b* in PLX3397 treated animals compared to controls, 6 hours after LPS injection. This was more pronounced in the PLX^hi^ treated group. After 24 hours, levels of *Tnf, Il1b, Il6* and *Ccl2* were lower in PLX^hi^ pretreated groups compared to control chow fed mice, although only reaching statistical significance for *Il1b* and a trend for *Il6*. Levels of *Ccl2* and *Il6* in PLX^lo^ pretreated animals Were also decreased at this timepoint, albeit not significantly. Of note, *Il1b* and *Il6* levels were already lower in PLX^hi^ treated animals at baseline (**Figure 5C** **;** **Table S5**). The reduction in KC numbers did not significantly impact LPS clearance at either timepoint (**Figure 5D** **;** **Table S7**).

In mice subjected to BDL, we observed an elevation of liver injury markers ALT and bilirubin, specifically in the PLX^hi^ treated group (**Figure S8A, B**). Additionally, we noted heightened bile infarct area in the PLX^hi^ treated group compared to untreated BDL mice (**Figure S8C, D**). BDL mice exhibit bacterial translocation. These bacteria are putatively cleared by KCs. Similar to our findings in the LPS model however, systemic endotoxemia as a result of bacterial translocation was similar in PLX3397 fed mice and chow fed BDL controls (**Figure S8E**). The hepatic mRNA expression levels of the macrophage marker *Csf1r* and the myeloid marker *Itgam*, both increased after BDL surgery, were significantly reduced when mice were treated with PLX^hi^. Of note, PLX^lo^ treated mice exhibited an intermediary phenotype, not significantly different from untreated BDL controls (**Figure S8F**). Finally, while the increase in hepatic *Tnf* levels after BDL was abrogated in PLX^hi^ treated mice, the levels of *Il6, Il1b* and *Ccl2*, although lower in PLX^hi^ treated mice, were not significantly different from untreated BDL controls. PLX^lo^ feeding did not affect the expression levels of these cyto/chemokines after BDL (**Figure S8G**).

### Levels of CNS infiltrating and circulating myeloid cells are reduced in a dose dependent manner upon PLX3397 treatment

**Figure 6:**
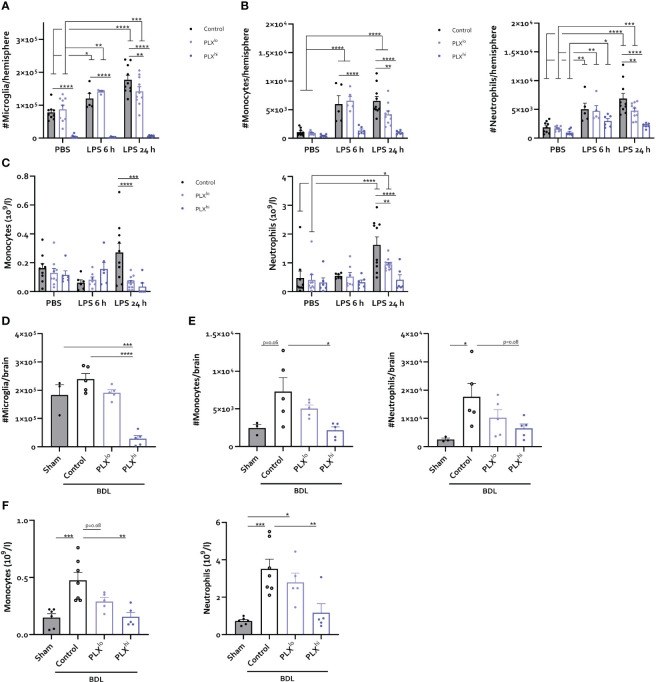
PLX3397 has dose dependent effects on CNS infiltrating and circulating myeloid cells. **(A–C)** Mice were treated with AIN76A chow containing no (control), low (75 ppm; PLX^lo^) or high (600 ppm; PLX^hi^) concentrations of PLX3397 for 7 days prior to injection of LPS (2.5 mg/kg, IP). Mice were sacrificed 6 and 24 hours after injection. Absolute number of microglia **(A)**, Ly6C^+^ Monocytes and Ly6G^+^ neutrophils **(B)** per hemisphere and levels of circulating monocytes and neutrophils **(C)**. Data are derived from 4 independent experiments with n= 5-10/group. **(D–F)** Mice were treated with AIN76A chow containing no (control), low (75 ppm; PLX^lo^) or high (600 ppm; PLX^hi^) concentrations of PLX3397 from 5 days after BDL surgery. Mice were sacrificed 14 days after induction surgery. Sham mice were used as controls. Absolute number of microglia **(D)**, Ly6C^+^ monocytes and Ly6G^+^ neutrophils **(E)** in the brain and levels of circulating monocytes and neutrophils **(F)**. Data are derived from 2 independent experiments with n= 3-7/group. * p<0.05, ** p<0.01, *** p<0.001, **** p<0.0001.

To investigate whether changes in the cellular immune response within the brain and periphery could account for the heightened response to peripheral inflammation, we examined levels of infiltrating immune cells in the CNS. Similar to our steady state experiments, only PLX^hi^ pretreated mice exhibited microglial ablation (**Figure 6A** **;** **Table S6**). Furthermore, no significant differences were detected in brain monocytes or neutrophils in PBS injected animals when comparing chow regimens (**Figure 6B** **;** **Table S6**). We observed that PLX^hi^ pretreated mice exhibited reduced levels of CNS-associated monocytes 6 and 24 hours after LPS injection, and reduced levels of CNS-associated neutrophils at 24 hours after LPS injections, compared to control chow fed animals. Interestingly, we observed that LPS induced microglial accumulation in control chow fed animals was diminished in PLX^lo^ pretreated animals. Additionally, PLX^lo^ too significantly reduced CNS accumulation of monocytes and neutrophils 24 hours after LPS, albeit to a lesser extent than in PLX^hi^ pretreated mice (**Figure 6A, B** **;** **Table S6**). In the chronic BDL model, which, in line with earlier observations (41, 49), is not associated with microglial proliferation (**Figure 6D**), we observed similar patterns regarding CNS monocyte and neutrophil numbers (**Figure 6E**). Considering these striking findings, we performed crude CBC analysis, which revealed that 24 hours after LPS and upon BDL induced hepatic and systemic inflammation, both PLX^lo^ and PLX^hi^ treated mice exhibited reduced levels of circulating monocytes and neutrophils compared to control chow fed mice. All effects were more pronounced when using a higher dose, and in BDL experiments, the monocyte/neutrophil reduction in PLX^lo^ treated animals was not significantly different from BDL animals fed control chow (**Figure 6C, F** **;** **Table S7**).

In line with our previous data (41), a small but non-significant increase in brain cDC counts was observed after BDL. This was not observed in response to LPS. Notably, a decreased cDC count, more pronounced with increasing dose, was detected in PBS-injected mice as well as in the PLX3397-treated BDL mice (**Figure S9B, G** **;** **Table S6**). Analysis of brain lymphoid cells did not reveal any brain influx in either disease model, nor consistent effects of PLX3397 (pre)treatment (**Figure S9A, F** **;** **Table S6**). Circulating lymphocytes were significantly reduced in PLX3397-preteated animals at either dose, 24 hours after LPS injection (**Figure S9C** **;** **Table S7**). In addition, BDL-induced lymphopenia was similar in all animals regardless of CSF1Ri treatment or dose (**Figure S9H**). As for platelets, we observed development of thrombopenia in LPS-injected mice, which was less severe in case of PLX3397 pretreatment at either dose after 24 hours (**Figure S9D**). In the BDL model, we rather observed thrombocytosis, which was reduced by PLX3397 treatment in a dose-dependent manner (**Figure S9I**). In line with data in the steady state experiments, RBC count was significantly lower in PLX^hi^ treated animals compared to untreated controls in either model and at all assessed timepoints (**Figure S9D** **;** **Table S7**). A trend towards decreasing RBC numbers was also found in PLX^lo^ treated animals after BDL (**Figure S9J**). Finally, LPS induced a transient increase in RBC count after 6 h, but only in control chow and PLX^lo^ fed mice (**Figure S9E** **;** **Table S7**).

### PLX induces myeloid and NK cell depletion in the bone marrow

**Figure 7:**
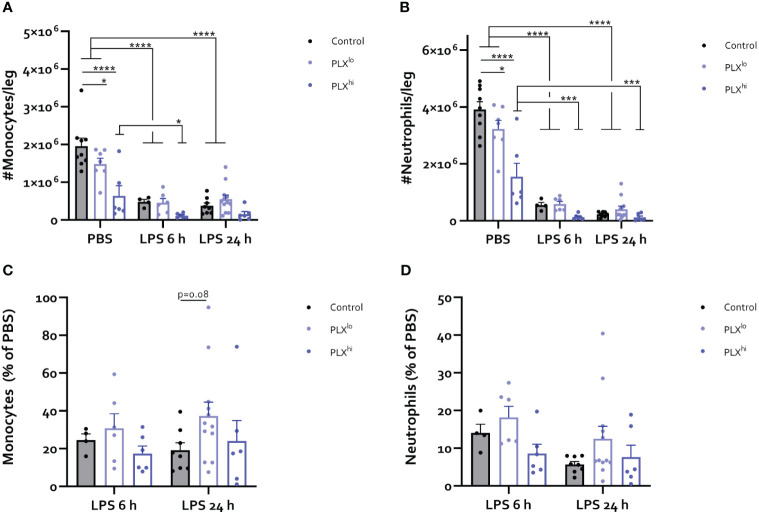
PLX3397 depletes bone marrow monocyte and neutrophil pool. Mice were treated with AIN76A chow containing no (control), low (75 ppm; PLX^lo^) or high (600 ppm; PLX^hi^) concentrations of PLX3397 for 7 days prior to injection of LPS (2.5 mg/kg, IP). Mice were sacrificed 6 and 24 hours after injection. **(A-D)** Absolute number of Ly6C^+^ monocytes **(A)** and Ly6G^+^ neutrophils **(B)** per leg. Relative amount of Ly6C+ monocytes **(C)** and Ly6G+ neutrophils **(D)** after LPS injection, compared to respective PBS controls. Data are derived from 4 independent experiments with n= 4-12/group. * p<0.05, ** p<0.01, *** p<0.001, **** p<0.0001.

Given the striking effects of PLX3397 treatment at both doses on myeloid cell levels in the brain and circulation, we sought to investigate whether these effects were mediated through PLX3397-induced bone marrow alterations. As illustrated in **Figure 7A-D**, LPS triggered recruitment of myeloid cells from the bone marrow, leading to extensive decreases in the number of monocytes and neutrophils. At either timepoint after LPS injection, we did not find significant differences between the PLX3397 treatment groups. However, PBS-injected PLX^hi^ pretreated mice exhibited a substantial reduction in the levels of both monocytes and neutrophils compared to control chow fed mice. Significant decreases in these cell types were also observed in the PLX^lo^ treated groups, although to a lesser extent, mirroring the 7-fold difference in ingested doses between both PLX3397 treated groups (**Figure 7A, B** **;** **Table S8**). To evaluate whether reduced monocyte and neutrophil numbers could additionally be attributable to reduced efflux from (and/or production of myeloid cells in) the bone marrow in response to an inflammatory stimulus, we assessed the relative amounts of monocytes and neutrophils in LPS *versus* PBS controls. A trend towards increased relative monocyte levels compared to control chow treated mice was detected in PLX^lo^ pretreated animals, 24 hours after LPS injection (**Figure 7C** **;** **Table S9**). Relative levels of neutrophils after LPS injection were comparable between PLX treatment groups (**Figure 7D** **;** **Table S9**). Evaluating lymphoid cells and cDCs, we observed reductions of absolute cell numbers in the bone marrow upon LPS injection, similarly indicating recruitment to the blood and other tissues (**Figure S10A, B**). Strikingly, in the PBS condition, we found reductions of not only cDC counts, but also NK cell counts in both PLX^lo^ and PLX^hi^ treated animals. Similar to our findings in monocytes and neutrophils, relative reduction of cell counts was not affected by PLX^hi^ pretreatment. In PLX^lo^ fed mice, trends for increased residual cell proportions after LPS injection were detected in NK cells, CD8^+^ T-cells and B-cells, only at 24 hours after LPS injection. This was not the case in cDCs or CD4^+^ T-cells (**Figure S10C, D** **;** **Table S9**).

## Discussion

The mononuclear phagocyte system (MPS) comprises a group of related cell types including monocytes, tissue macrophages, classical dendritic cell (cDCs) and their progenitors. These cells share the expression of colony-stimulating factor 1 receptor (CSF1R), a tyrosine kinase that plays a crucial role in maturation, proliferation and survival, regulated by its ligands IL-34 and CSF1 (50). Consequently, inhibiting CSF1R signalling can have profound effects on MPS cell abundance and function (15). Blood-brain barrier (BBB) penetrant small molecule CSF1R inhibitors (CSF1Ri) are of particular interest for the study of microglia, the brain resident mononuclear phagocyte. Due to the reversible nature of the inhibition, they make versatile modulation possible (12). However, it is important to recognize the impact of these compounds on non-microglial cells. Unfortunately, the examination of such effects is often inadequate, despite their potential relevance in disease settings when using BBB penetrant CSF1Ri. A comprehensive understanding of these compounds’ broader implications is vital for accurately interpreting experimental outcomes.

Here, we investigated the effects of PLX3397, a commonly used compound for studying microglia in health and disease, on the MPS in the brain, liver, circulation and bone marrow. We confirmed previous finding that a high dose of PLX3397 (600 ppm in chow; PLX^hi^) depleted microglia within 7 days of administration, while a lower dose (75 ppm; PLX^lo^) had no effect on microglial cell numbers (17, 18). In addition, we did not find an influx of monocytes into the brain upon pharmacological microglial depletion (17, 45). Interestingly, the impact of PLX3397 extended beyond the central nervous system (CNS) for both doses, leading to a dose-dependent reduction in Kupffer cells (KCs) in the liver, consistent with reversible transaminase increases seen in patients (44, 51) and findings in mice treated with PLX5622, a compound derived from PLX3397, but with higher specificity for the CSF1R over related kinases Fms like tyrosine kinase 3 (Flt3) and c-Kit (25, 30, 52). Confirming data from general macrophage depletion paradigms, ALT elevations did not coincide with clear histopathological liver injury, suggesting a role for KCs in transaminase clearance (53). Although KC depletion is usually associated with a rapid influx of bone marrow-derived monocytes and the development of monocyte-derived KCs (26, 46), we did not observe changes in hepatic or circulating monocyte numbers, suggesting defective recruitment from the bone marrow. Indeed, we found that treatment with PLX3397 induces dose-dependent reductions in bone marrow monocyte numbers, even at a low dose, indicating that there is no dose can selectively deplete microglia without significant bone marrow toxicity. Similarly, PLX5622 reduced the number of bone marrow-derived inflammatory monocytes in Western Nile virus encephalitis without affecting microglial cell counts at a dose of 200 ppm (31). These findings highlight the complexities of using PLX3397 and PLX5622 in microglial studies and underscore the need for careful consideration of their off-target effects on peripheral immune cells.

While under steady state conditions, the impact of PLX3397 on the peripheral MPS may seem less relevant, our data indicate that this is not the case in pathological conditions. In the LPS endotoxemia model, we observed increased mortality in mice upon PLX3397 pretreatment, which is consistent with findings of prolonged sickness behaviour after LPS injection (35) and increased mortality in polymicrobial sepsis (36) in mice pretreated with PLX5622. Additionally, the temperature response was exacerbated. The body weight drop was slightly less pronounced in the PLX^hi^ pretreated group, possibly due to a lower absolute starting body weight. Effects on KCs could only partly account for this increased mortality, as LPS-induced ALT elevations were limited and only slightly exacerbated in high dose pretreated mice. Moreover, LPS clearance was similar in all treatment groups and effects on the hepatic inflammatory response were relatively limited. The inflammatory response in the brain was also decreased, but only in the high dose treatment group, again only partly accounting for the altered phenotype. Interestingly however, at both doses, we found evidence of impaired recruitment of monocytes and neutrophils to the circulation and the brain, linked to reduced bone marrow pool for these cell types. The fact that this was detectable in the low dose treatment group indicates this is a compound-specific effect which can additionally explain the intermediary increase of mortality after LPS injection in the low dose treatment group. Additionally, this indicates that in this set-up, it is impossible to distinguish the role of microglial presence on the myeloid cell infiltration to the brain.

Interestingly, we observed PLX3397-associated reductions in several unexpected cell types. In case of monocytes and cDCs, reduced numbers can be explained by their CSF1R expression (29, 30, 50, 54) and are in line with previous reports using PLX3397 and PLX5622 (28–31). In contrast, neutrophils are believed to not express CSF1R protein, and therefore should not be affected by its inhibition (50, 55). Although one study has reported a similar effect (52), this contradicts most reports using PLX5622 (31, 56), CSF1R blocking monoclonal antibodies (mAbs), and *Csf1r* ^ΔFIRE/ΔFIRE^ mice (16, 57). The discrepancy between PLX3397 and PLX5622 suggests that non-CSF1R-related toxicity, which is more likely in PLX3397, might be responsible for these effects, similar to what has been described for oligodendrocyte precursors (58). The reduction in NK cells, while unexpected, aligns with previous reports (28, 29) and might be linked with decreased myeloid cell-derived IL-15, which is necessary for NK cell survival (59).

The finding of increased mortality after LPS when pretreating with PLX3397 was surprising. Monocyte/macrophage and NK cell reductions or depletion experiments have rather been associated with an attenuated response to the LPS model of endotoxemia, which lacks microbial infection (60, 61). Furthermore, the LPS-induced inflammatory response, which putatively drives organ dysfunction and mortality, was not exacerbated, but reduced in our experiments. As neutrophil depletion has been shown to increase mortality in LPS-induced endotoxemia (62), we speculate that this effect on neutrophils partially explains the exacerbated phenotype we observe despite the attenuated cytokine response in PLX^hi^ mice. We additionally can’t exclude that effects of PLX3397 on microglia directly affects the systemic inflammatory response, dampening the systemic inflammation feedback loop. Future research could elucidate the operant mechanism of this exacerbated phenotype.

We further confirmed our findings in the bile duct ligation (BDL) model, which is characterized by liver inflammation with secondary microglial activation and influx of immune cells in the brain (41, 49). Here, we observed significant worsening of liver injury in the PLX^hi^ treated group after BDL, consistent with non-microglial effects of CSF1R inhibition and showing that the PLX off-target effects depend on the disease model studied. This exacerbated liver injury is consistent with findings using other non-specific monocyte/macrophage depletion methods (63, 64), but not KC-specific depletion (65), again implying broad-spectrum effects of PLX3397. Differences in the effect of PLX3397 on LPS/BDL-induced platelet and lymphocyte changes in the blood further underscore that the disease model partly dictates the off-target effects of PLX3397. However, we again observed reduced accumulation of myeloid cells in the BDL brain and circulation at both doses, suggesting that this mechanism is of universal importance when using PLX3397 at any dose in the context of peripheral inflammation.

Our findings offer crucial insights into how bone marrow alterations may play a role in the observed dynamics of myeloid cell responses when using PLX3397-dependent microglia depletion in combination with peripheral inflammation. Of note, using crude CBC analyses, differences were only noticeable when employing an inflammatory trigger, possibly explaining why some studies employing limited evaluation in non-inflammatory conditions could not detect changes in immune cell populations (23, 24). Furthermore, we are the first to perform extensive phenotyping of the bone marrow mature immune cell pool using PLX3397. Our data confirms findings from studies utilizing PLX5622, showing significant impairment of bone marrow monocyte production (30, 31). Our data collectively suggest that PLX3397 can directly or indirectly affect the cell survival of myeloid and NK cells, thus compromising their ability to cope with a proinflammatory stimulus. It’s worth nothing that PLX3397 is known to inhibit kinases similar to CSF1R, namely c-kit and Flt3 (66). These kinases are necessary for normal hematopoiesis and inhibition might consequently contribute to the decreased myeloid cell pool in the bone marrow. Interestingly, a recent report indicates that after 14 days of PLX^hi^ treatment resulted in the expansion, rather than supression, of bone marrow common myeloid and granulocyte-monocyte progenitors, which give rise to monocytes, neutrophils (45). However, it’s worth noting that neutrophil depletion can trigger ‘emergency granulopoiesis’, leading to progenitor expansion (67). This suggests that there is an insufficient ‘emergency hematopoiesis’ response to cope with the reduced mature cell pool, which could explain our seemingly contradictory findings compared to those of Hohsfield et al. ( 45).

Furthermore, our data shed light on the use of PLX^lo^ as a ‘peripheral control’. While PLX^lo^ treatment in some regards produced similar effects on liver, blood and bone marrow as PLX^hi^, these were consistently less pronounced. For instance, only PLX^hi^ treated mice showed exacerbated liver injury after BDL. Additionally, PLX^lo^ treatment seemed effective in inhibiting LPS-induced microglial accumulation, similar to other small molecule CSF1R kinase inhibitors (68). Moreover, astrocytic glial fibrillary acidic protein (*Gfap)* upregulation and decreased ionized calcium-binding adapter molecule 1 (IBA1) stained area in PLX^lo^ treated animals might be related to the development of a phagocytic astrocyte phenotype aimed at clearing microglial debris (69, 70), and suggests some degree of microglial cell death even at this dose. It is worth noting that PLX^lo^ treatment did not affect BBB permeability, but we did observe increased permeability of the blood-CSF barrier, which may be attributed to effects on choroid plexus (ChP) stromal macrophages or epiplexus cells. Hence, PLX^lo^’s impact on the brain cannot be overlooked, while the peripheral effects seen at higher doses are also not entirely replicated. In summary, PLX^lo^ can at best be considered as a partial peripheral control.

An important limitation of our study concerns the dosing of PLX3397. Typically, it is administered in chow at a dose of 290 ppm, about half of the PLX^hi^ dose we employed. At 290 ppm, microglial depletion is achieved within 21 days after the chow administration (17) although differences are reported depending on the chow formulation and animal gender (28). In contrast, the PLX^hi^ dose employed in our study generally leads to rapid and more extensive microglia depletion within 7 days (45, 71). Some of the unique effects observed at the PLX^hi^ dose, such as the reduction in red blood cells (RBCs), might be linked to c-kit inhibition rather than CSF1R blockade. This suggests that the PLX^hi^ dose likely induces relevant effects on hematopoiesis through c-Kit inhibition. These hematopoietic effects have not been observed with the standard 290 ppm dose but have been reported at a dose of 400 ppm (28).

In summary, our data demonstrate that PLX3397 has significant effects on the MPS in the brain, peripheral tissues, and bone marrow. The observed neutrophil depletion and RBC reduction also suggest non-CSF1R mediated effects when using this compound. Giving these off-target effects, results obtained using systemically administered PLX3397 (and PLX5622) for studying microglia in CNS pathology should be interpreted with caution, specifically of CNS disease is driven by peripheral inflammation. However, in other neuroinflammatory diseases, it is crucial to carefully characterize these effects to distinguish microglial-specific effects from generalized CSF1R inhibition effects. Considering the recent identification of the skull bone marrow as a separate important contributor to CNS-infiltrating myeloid cells compared to the blood, effects of PLX3397/PLX5622 on the skull bone marrow should also be evaluated (72, 73). Lower doses of PLX3397, which fail to deplete microglia, can at best serve as partial controls, and their potential (in)direct effects on the CNS should be taken into consideration. Researchers may explore alternative strategies, such as non-BBB penetrant compounds or mAbs, to control for peripheral effects, while considering differences in specificity and activity against CSF1R and related kinases. Another potential approach involves repeated intracerebroventricular injection of PLX3397, as this has been shown to deplete microglia without affecting peripheral tissues or bone marrow (29, 74). Further efforts should be focused on developing long-term and modulatable depletion and repopulation paradigms that are truly microglia-specific to advance our understanding of microglial function in health and disease.

## Data availability statement

The raw data supporting the conclusions of this article will be made available by the authors, without undue reservation.

## Ethics statement

The animal study was approved by Animal Ethics Committee, Faculty of Sciences, Ghent University, Ghent, Belgium. The study was conducted in accordance with the local legislation and institutional requirements. All experiments complied with the current laws of Belgium (Law of 14. August 1986 related to protection and welfare of animals) and EU directive 2010/63/EU, and were approved by the animal ethics committee of the Faculty of Sciences, Ghent University (EC 2022-070, EC2023-034, EC2023-018).

## Author contributions

WC: Writing – original draft, Conceptualization, Formal Analysis, Investigation, Methodology, Visualization. DV: Investigation, Writing – review & editing, Methodology. GV: Investigation, Writing – review & editing. EW: Investigation, Writing – review & editing. LV: Investigation, Writing – review & editing. LA: Investigation, Writing – review & editing. FD: Investigation, Writing – review & editing. CS: Methodology, Writing – review & editing. AG: Funding acquisition, Writing – review & editing. CV: Funding acquisition, Methodology, Supervision, Writing – original draft. LV: Conceptualization, Investigation, Methodology, Supervision, Writing – original draft. RV: Conceptualization, Funding Acquisition, Methodology, Supervision, Writing – original draft.

## Supplementary material

Supplementary File 1 Detailed protocols for behavioural evaluation

Supplementary Figure 1 Gating strategy for brain cells. **(A)** Strategy for immune cell phenotyping in brain of sham/BDL mice and PBS/LPS mice. * denotes strategy only employed in BDL/sham mouse brains. **(B)** Strategy for isolation of astrocytes. Cells are pregated on live singlets.

Supplementary Figure 2 Gating strategy for bone marrow cells.

Supplementary Figure 3 Gating strategy for isolation of liver immune cells.

Supplementary Figure 4 High dose PLX3397 induces anorexia and reduced activity in the open field. Mice were treated with AIN76A chow containing no (control), low (75 ppm; PLX^lo^) or high (600 ppm; PLX^hi^) concentrations of PLX3397 for 7 days prior to sacrifice and analysis. **(A)** Body weight evolution after diet start. Data derived from 2 independent repeats with n=10-11/group. **(B)** Average daily food intake 7 days after diet start. Data derived from 6 independent repeats with n=11-16/group. **(C)** Distance travelled in the open field test 6 days after diet start compared to baseline levels and time spent in the center of the open field. **(D)** Average beam traversal time 6 days after diet start compared to baseline. Behaviour data are derived from 2 independent repeats with n=10-11/group.

Supplementary Figure 5 PLX3397 affects astrocytes and the blood-CSF barrier. Mice were treated with AIN76A chow containing no (control), low (75 ppm; PLX^lo^) or high (600 ppm; PLX^hi^) concentrations of PLX3397 for 7 days prior to sacrifice and analysis. **(A-C)** Representative flow cytometry plots **(A)**, absolute cell counts of ACSA2+ astrocytes **(B)** and *Gfap* mRNA levels in sorted astrocytes **(C)**. **(D-I)** Representative images and quantification of GFAP **(D, E)**, Olig2 **(F, G)** and NeuN **(H, I)** staining in hippocampus. Data are derived from a single experiment with n=5/group. **(F)** Relative barrier permeability of the BBB and the blood-CSF barrier as assessed by fluorescence intensity in cortex, hippocampus, cerebellum and CSF after IV injection of 4kDa FITC-Dextran. Data are derived from a single experiment with n=6-7/group.

Supplementary Figure 6 PLX3397 does not affect body weight or temperature response to an LD_0_ dose of LPS. Mice were treated with AIN76A chow containing no (control), low (75 ppm; PLX^lo^) or high (600 ppm; PLX^hi^) concentrations of PLX3397 for 7 days prior to injection of LPS (2.5 mg/kg, IP). Mice were sacrificed 6 and 24 hours after injection. **(A-B)** Body temperature **(A)** and relative body weight **(B)**. Data are derived from 4 independent repeats with n=8-13/group.

Supplementary Figure 7 PLX3397 exacerbates the response to BDL. **(A)** Experimental set-up. Mice were treated with AIN76A chow containing no (control), low (75 ppm; PLX^lo^) or high (600 ppm; PLX^hi^) concentrations of PLX3397 from 5 days after BDL surgery. Mice were sacrificed 14 days after induction surgery. Sham mice were used as controls. **(B)** Survival graphs. **(C)** Body temperature at sacrifice. Data are derived from 2 independent repeats with n=5-7/group.

Supplementary Figure 8 High dose PLX3397 exacerbates liver injury in BDL mice. Mice were treated with AIN76A chow containing no (control), low (75 ppm; PLX^lo^) or high (600 ppm; PLX^hi^) concentrations of PLX3397 from 5 days after BDL surgery. Mice were sacrificed 14 days after induction surgery. Sham mice were used as controls. **(A-B)** Plasma levels of ALT **(A)** and bilirubin **(B)**. **(C-D)** Bile infarctions with representative images of H&E stained sections (C) and quantification of bile infarct area **(D)**. Scale bar represents 100 µm. **(E)** Plasma LPS levels. **(F-G)** Hepatic expression level of the macrophage related gene *Csf1r*, myeloid related gene *Itgam* **(F),** inflammatory cytokines (*Tnf, Il1b, Il6*) and chemokines (*Ccl2*) **(G)**. All data are derived from 3 independent experiments with n=2-11/group depending on the outcome measure.

Supplementary Figure 9 Effects of PLX3397 on lymphoid cells, cDCs, red blood cells and platelets are model dependent. **(A-E)** Mice were treated with AIN76A chow containing no (control), low (75 ppm; PLX^lo^) or high (600 ppm; PLX^hi^) concentrations of PLX3397 for 7 days prior to injection of LPS (2.5 mg/kg, IP). Mice were sacrificed 6 and 24 hours after injection. Absolute counts of CD4+ T-cells, CD8+ T-cells, NK cells **(A)** and cDCs per hemisphere **(B)**. Circulating levels of lymphocytes **(C)**, platelets **(D)** and RBCs **(E)**. Data are derived from 4 independent experiments with n= 5-10/group. **(F-J)** Mice were treated with AIN76A chow containing no (control), low (75 ppm; PLX^lo^) or high (600 ppm; PLX^hi^) concentrations of PLX3397 from 5 days after BDL surgery. Mice were sacrificed 14 days after induction surgery. Sham mice were used as controls. Absolute counts of T-cells, B-cells **(F)** and cDCs per brain **(G)**. Circulating levels of lymphocytes **(H)**, platelets **(I)** and RBCs **(J)**. Data are derived from 2 independent experiments with n= 3-7/group.

Supplementary Figure 10 PLX3397 depletes cDCs and NK cells in the bone marrow. Mice were treated with AIN76A chow containing no (control), low (75 ppm; PLX^lo^) or high (600 ppm; PLX^hi^) concentrations of PLX3397 for 7 days prior to injection of LPS (2.5 mg/kg, IP). Mice were sacrificed 6 and 24 hours after injection. **(A, B)** Absolute number of CD4+ T-cells, CD8+ T-cells, B-cells, NK cells **(A)** and cDCs per leg **(B)**. **(C, D)** Relative amount of CD4+ T-cells, CD8+ T-cells, B-cells, NK cells **(C)** and cDCs **(D)** after LPS injection, compared to respective PBS controls. Data are derived from 4 independent experiments with n= 4-12/group.

Supplementary Table 1 Antibodies used for flow cytometry

Supplementary Table 2 Primer sequences used for qPCR

Supplementary Table 3 Antibodies used for immunofluorescence

Supplementary Table 4 ANOVA table of brain gene expression levels in PBS/LPS injected mice pretreated with different doses PLX3397 chow

Supplementary Table 5 ANOVA table of liver gene expression levels in PBS/LPS injected mice pretreated with different doses PLX3397 chow

Supplementary Table 6 ANOVA table of absolute counts of brain immune cells in PBS/LPS injected mice pretreated with different doses PLX3397 chow

Supplementary Table 7 ANOVA table of absolute counts of circulating cells, plasma ALT and LPS in PBS/LPS injected mice pretreated with different doses PLX3397 chow

Supplementary Table 8 ANOVA table of absolute counts of bone marrow immune cells in PBS/LPS injected mice pretreated with different doses PLX3397 chow

Supplementary Table 9 ANOVA table of relative amounts bone marrow immune cells LPS injected mice pretreated with different doses PLX3397 chow, compared to PBS controls
