# Supplementary figures and images for: Limitations of PLX3397 as a microglial investigational tool: peripheral and off-target effects dictate the response to inflammation
Source: Front Immunol. 2023 Nov 22;14:1283711. doi: 10.3389/fimmu.2023.1283711 (PMC10703484; doi:10.3389/fimmu.2023.1283711)

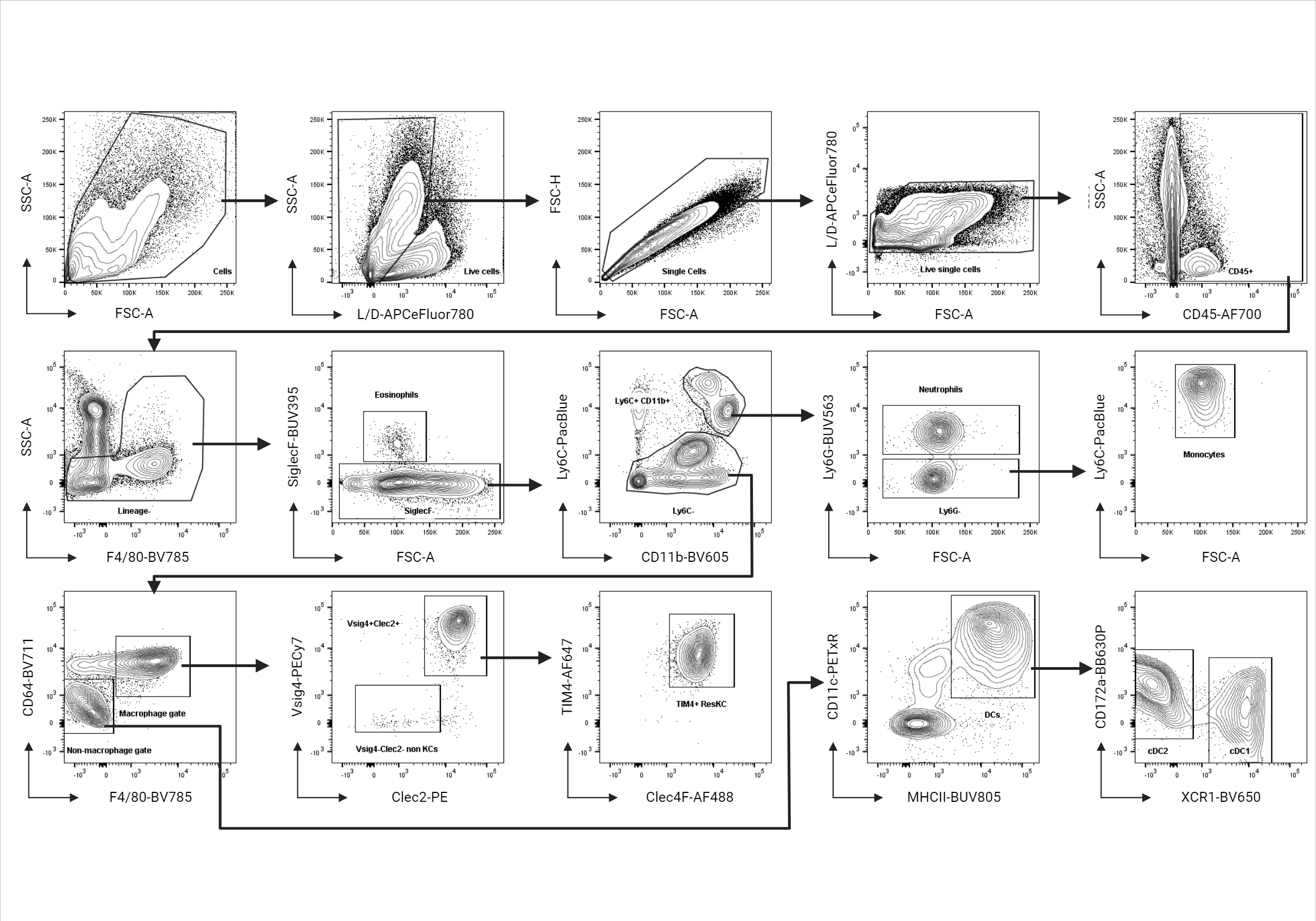

Supplement: Supplementary File 1 — Detailed protocols for behavioural evaluation [file Image_1.jpeg]

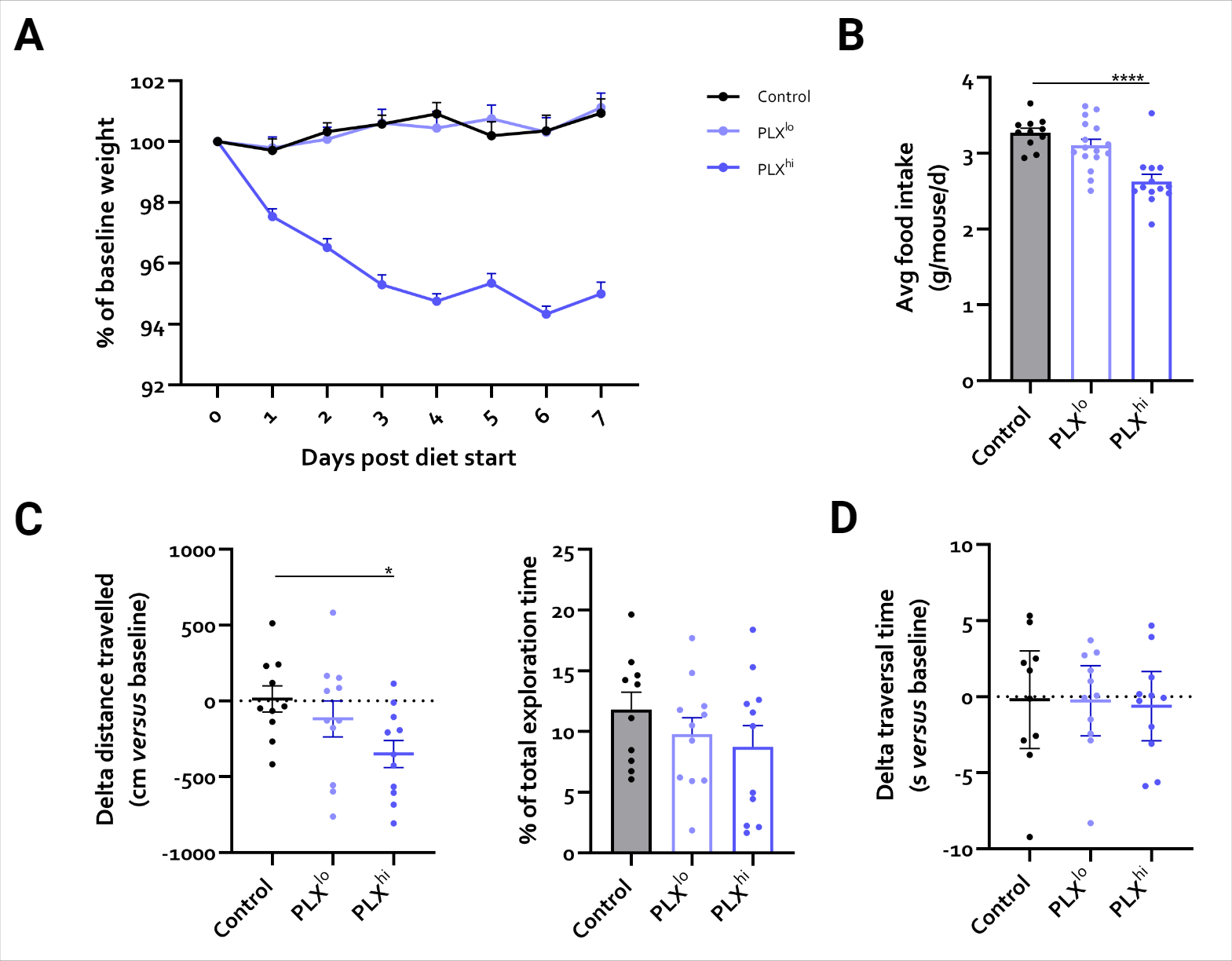

Supplement: Supplementary Figure 2 — Gating strategy for bone marrow cells. [file Image_2.jpeg]

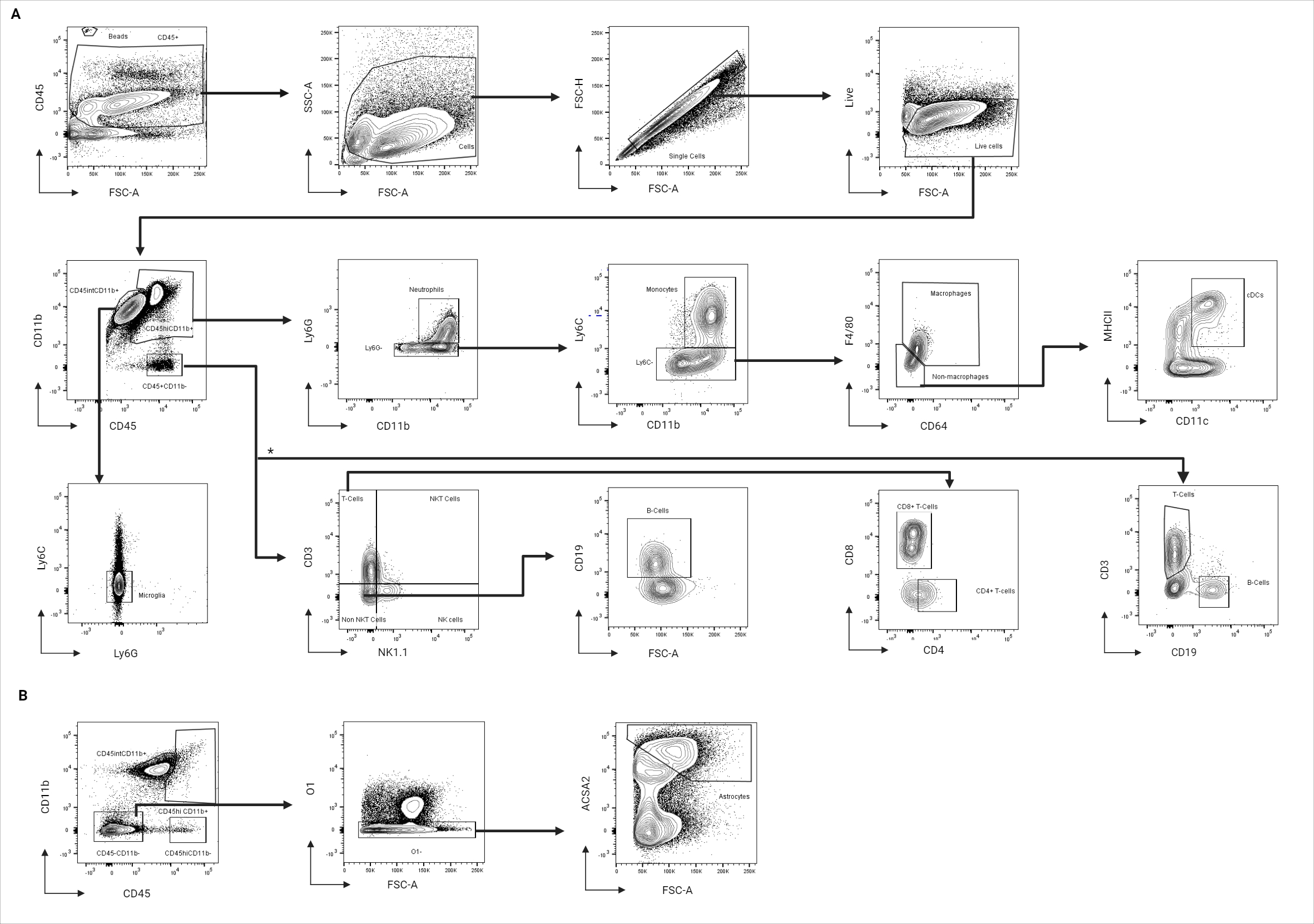

Supplement: Supplementary Figure 3 — Gating strategy for isolation of liver immune cells. [file Image_3.jpeg]

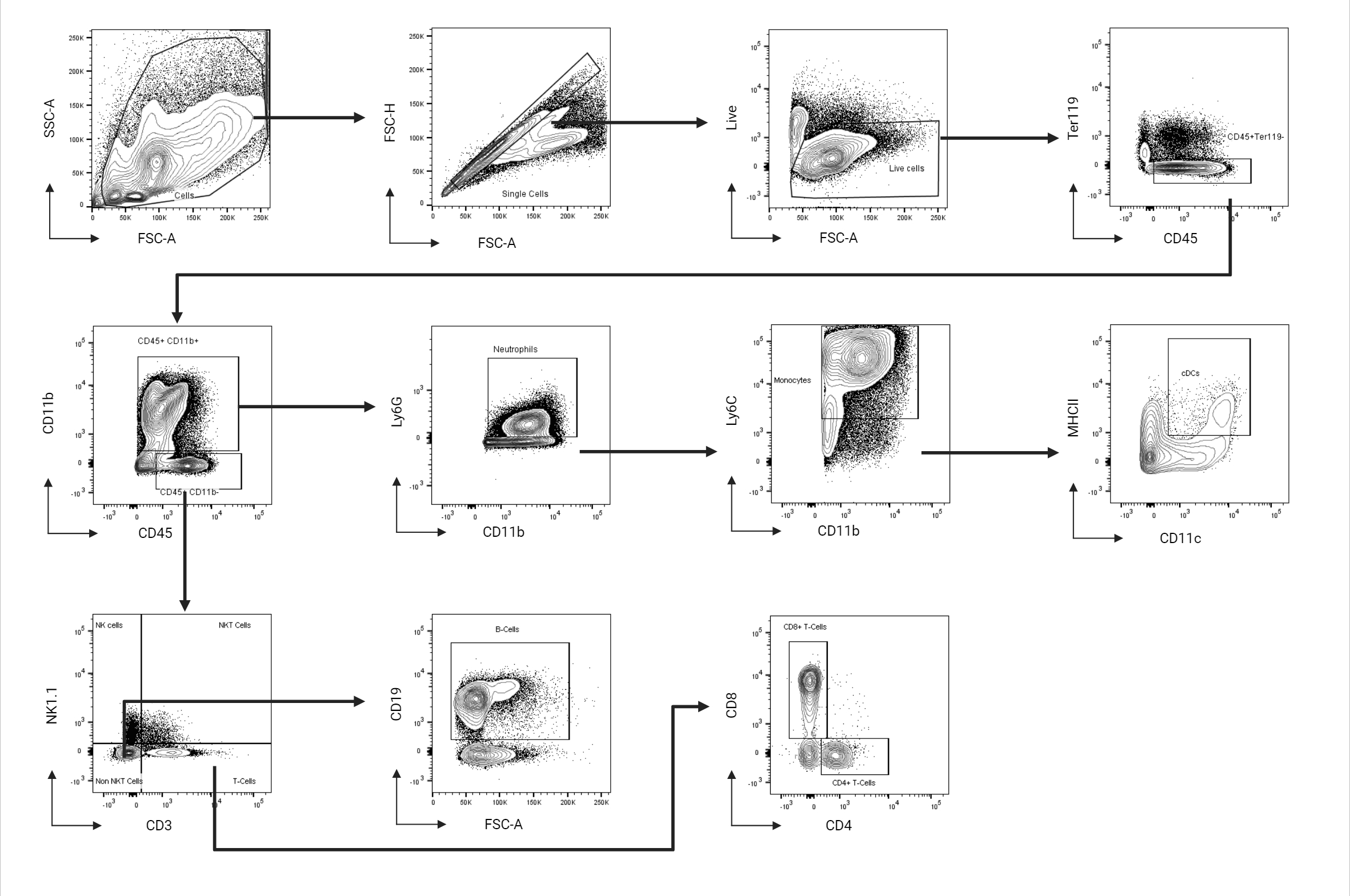

Supplement: Supplementary Figure 4 — High dose PLX3397 induces anorexia and reduced activity in the open field. Mice were treated with AIN76A chow containing no (control), low (75 ppm; PLXlo) or high (600 ppm; PLXhi) concentrations of PLX3397 for 7 days prior to sacrifice and analysis. (A) Body weight evolution after diet start. Data derived from 2 independent repeats with n=10-11/group. (B) Average daily food intake 7 days after diet start. Data derived from 6 independent repeats with n=11-16/group. (C) Distance travelled in the open field test 6 days after diet start compared to baseline levels and time spent in the center of the open field. (D) Average beam traversal time 6 days after diet start compared to baseline. Behaviour data are derived from 2 independent repeats with n=10-11/group. [file Image_4.jpeg]

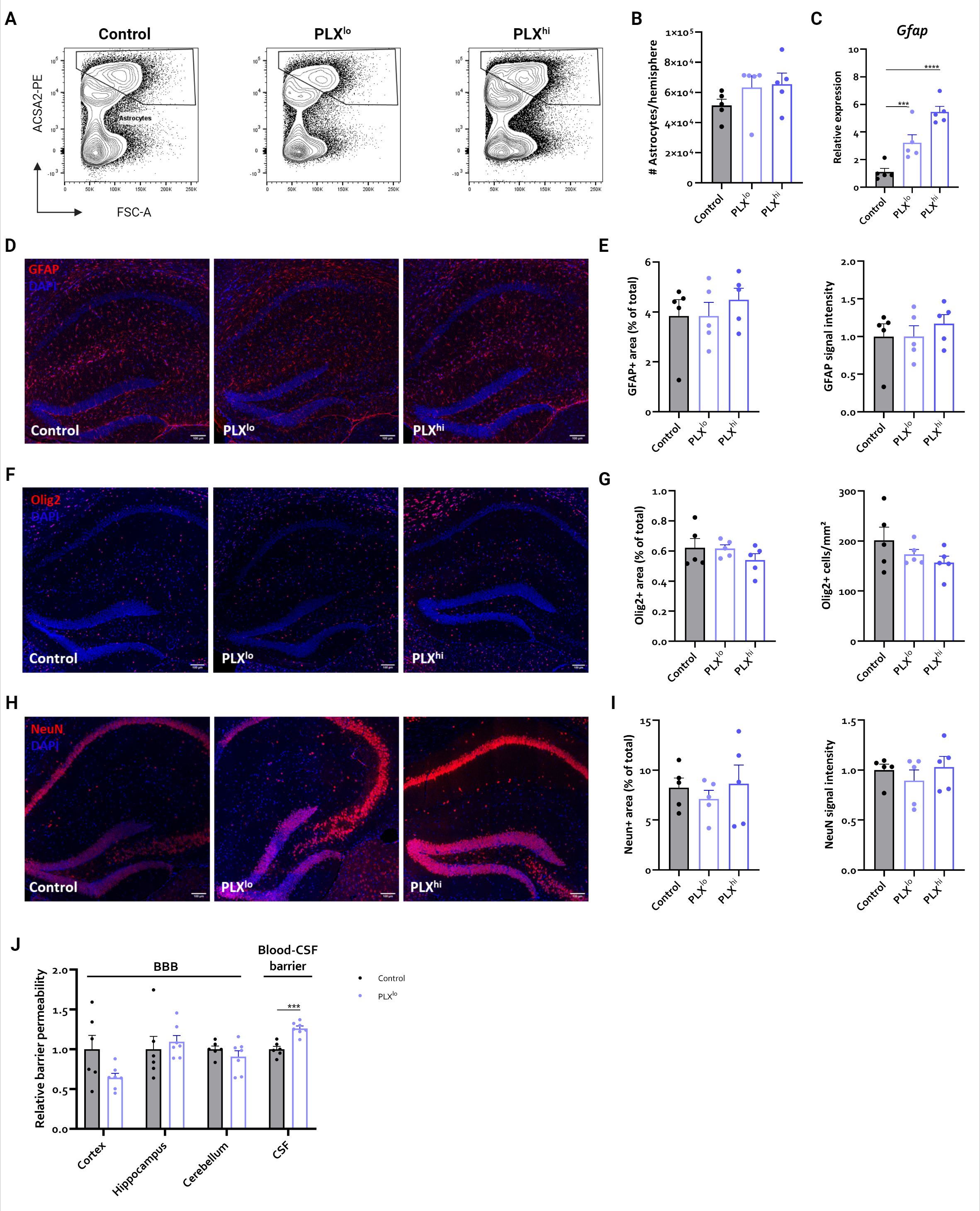

Supplement: Supplementary Figure 5 — PLX3397 affects astrocytes and the blood-CSF barrier. Mice were treated with AIN76A chow containing no (control), low (75 ppm; PLXlo) or high (600 ppm; PLXhi) concentrations of PLX3397 for 7 days prior to sacrifice and analysis. (A-C) Representative flow cytometry plots (A), absolute cell counts of ACSA2+ astrocytes (B) and Gfap mRNA levels in sorted astrocytes (C). (D-I) Representative images and quantification of GFAP (D, E), Olig2 (F, G) and NeuN (H, I) staining in hippocampus. Data are derived from a single experiment with n=5/group. (F) Relative barrier permeability of the BBB and the blood-CSF barrier as assessed by fluorescence intensity in cortex, hippocampus, cerebellum and CSF after IV injection of 4kDa FITC-Dextran. Data are derived from a single experiment with n=6-7/group. [file Image_5.jpeg]

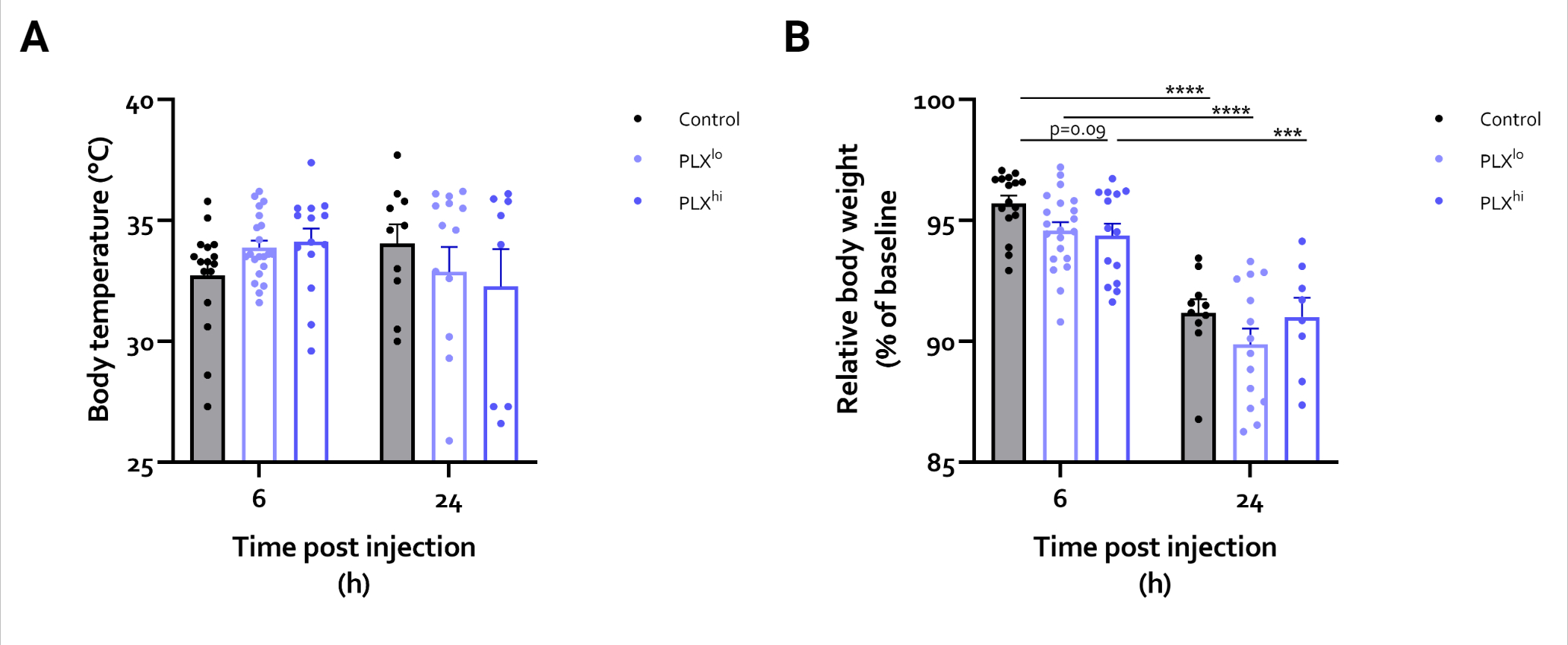

Supplement: Supplementary Figure 6 — PLX3397 does not affect body weight or temperature response to an LD0 dose of LPS. Mice were treated with AIN76A chow containing no (control), low (75 ppm; PLXlo) or high (600 ppm; PLXhi) concentrations of PLX3397 for 7 days prior to injection of LPS (2.5 mg/kg, IP). Mice were sacrificed 6 and 24 hours after injection. (A-B) Body temperature (A) and relative body weight (B). Data are derived from 4 independent repeats with n=8-13/group. [file Image_6.jpeg]

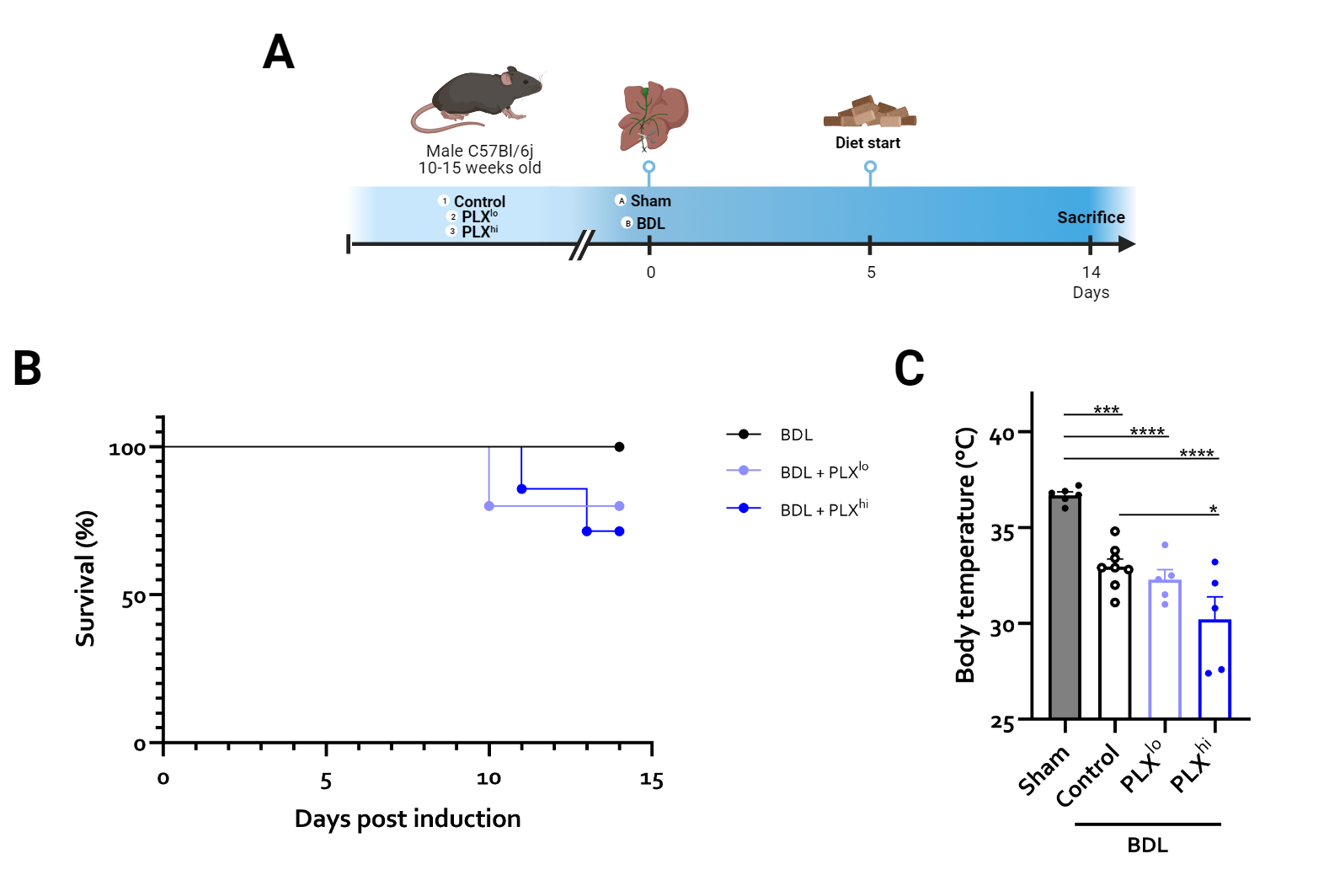

Supplement: Supplementary Figure 7 — PLX3397 exacerbates the response to BDL. (A) Experimental set-up. Mice were treated with AIN76A chow containing no (control), low (75 ppm; PLXlo) or high (600 ppm; PLXhi) concentrations of PLX3397 from 5 days after BDL surgery. Mice were sacrificed 14 days after induction surgery. Sham mice were used as controls. (B) Survival graphs. (C) Body temperature at sacrifice. Data are derived from 2 independent repeats with n=5-7/group. [file Image_7.jpeg]

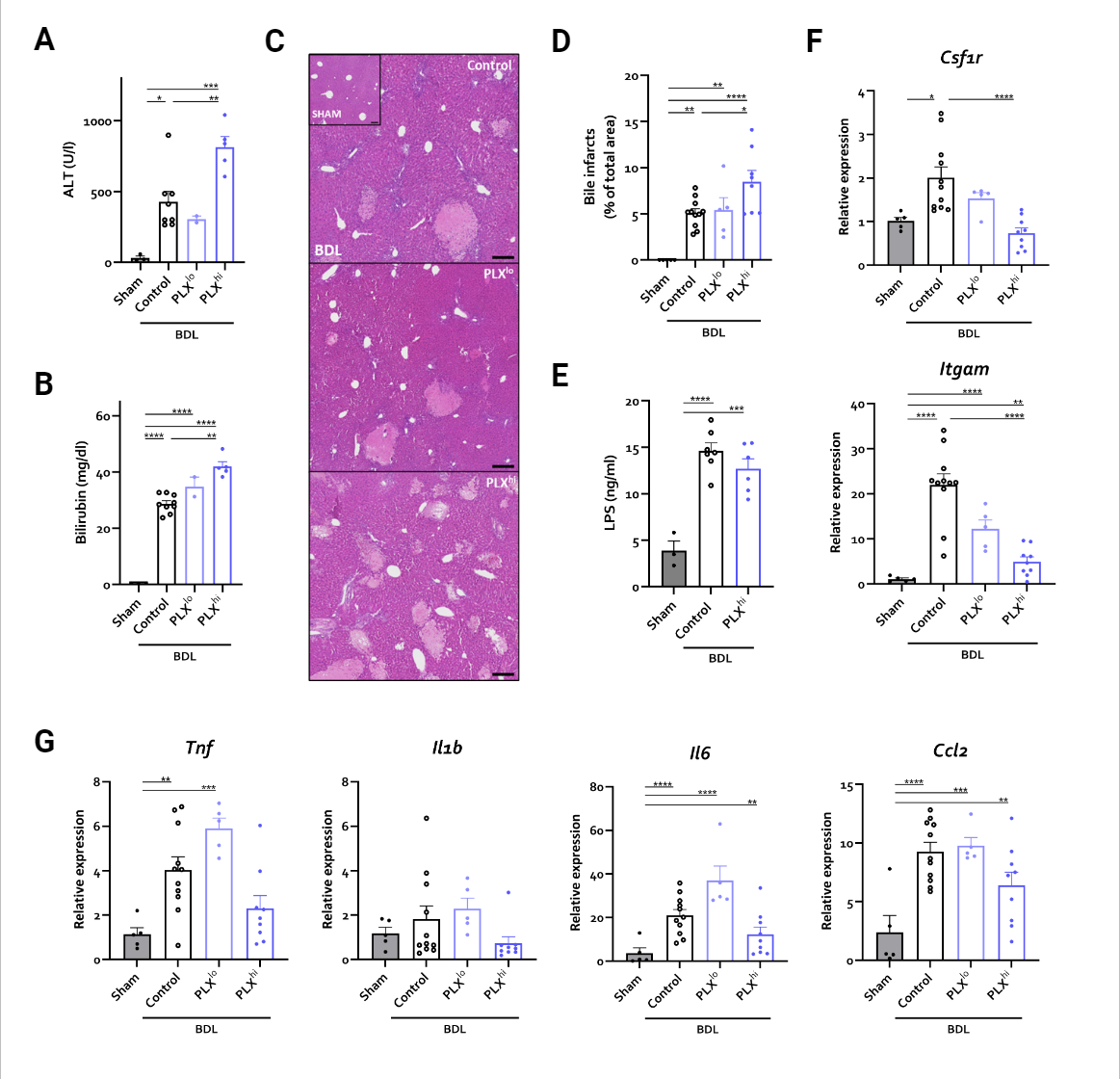

Supplement: Supplementary Figure 8 — High dose PLX3397 exacerbates liver injury in BDL mice. Mice were treated with AIN76A chow containing no (control), low (75 ppm; PLXlo) or high (600 ppm; PLXhi) concentrations of PLX3397 from 5 days after BDL surgery. Mice were sacrificed 14 days after induction surgery. Sham mice were used as controls. (A-B) Plasma levels of ALT (A) and bilirubin (B). (C-D) Bile infarctions with representative images of H&E stained sections (C) and quantification of bile infarct area (D). Scale bar represents 100 µm. (E) Plasma LPS levels. (F-G) Hepatic expression level of the macrophage related gene Csf1r, myeloid related gene Itgam (F), inflammatory cytokines (Tnf, Il1b, Il6) and chemokines (Ccl2) (G). All data are derived from 3 independent experiments with n=2-11/group depending on the outcome measure. [file Image_8.jpeg]

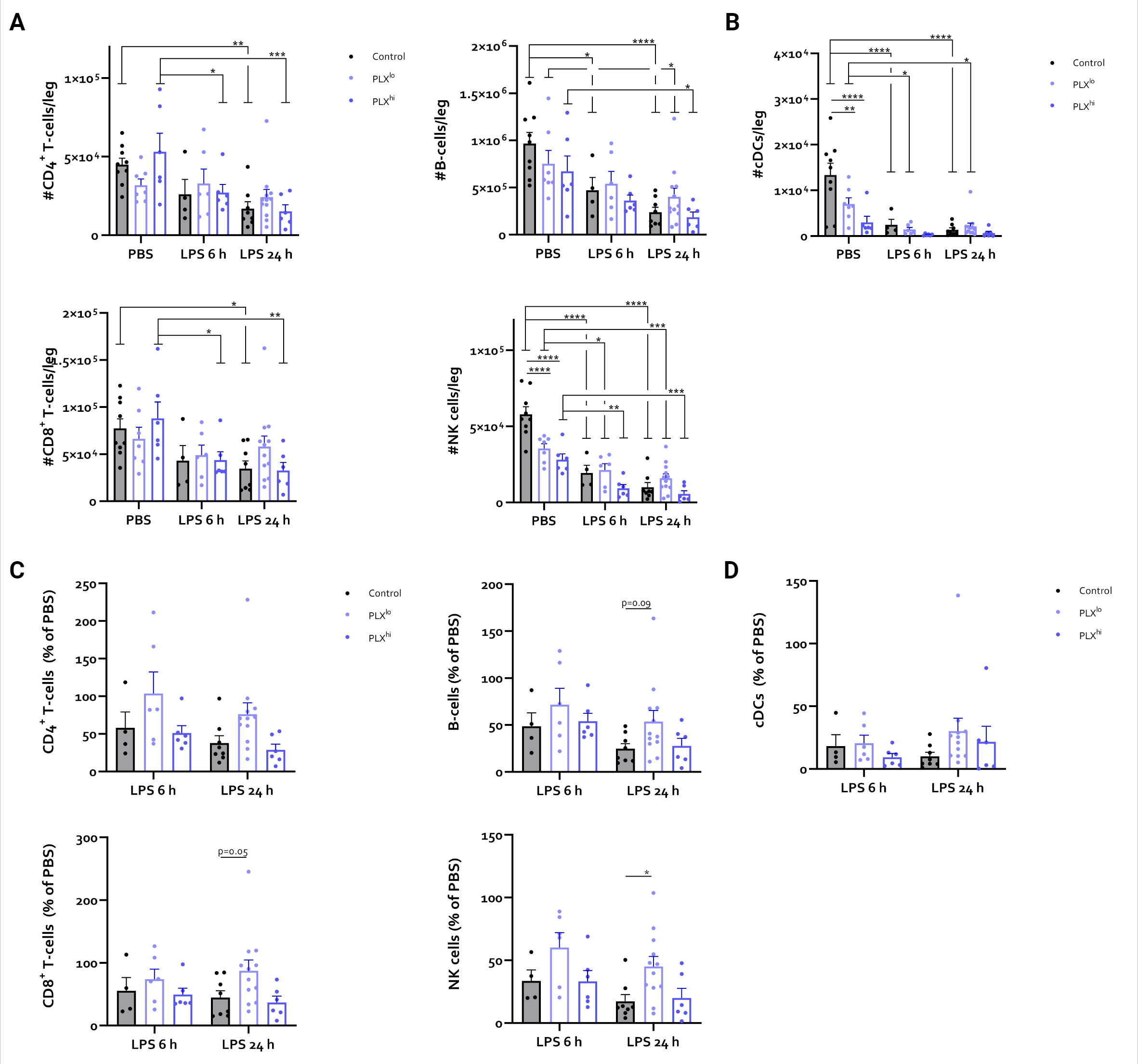

Supplement: Supplementary Figure 10 — PLX3397 depletes cDCs and NK cells in the bone marrow. Mice were treated with AIN76A chow containing no (control), low (75 ppm; PLXlo) or high (600 ppm; PLXhi) concentrations of PLX3397 for 7 days prior to injection of LPS (2.5 mg/kg, IP). Mice were sacrificed 6 and 24 hours after injection. (A, B) Absolute number of CD4+ T-cells, CD8+ T-cells, B-cells, NK cells (A) and cDCs per leg (B). (C, D) Relative amount of CD4+ T-cells, CD8+ T-cells, B-cells, NK cells (C) and cDCs (D) after LPS injection, compared to respective PBS controls. Data are derived from 4 independent experiments with n= 4-12/group. [file Image_10.jpeg]
